# Feline microRNAome in ovary and testis: Exploration of *in-silico* miRNA-mRNA networks involved in gonadal function and cellular stress response

**DOI:** 10.3389/fgene.2022.1009220

**Published:** 2022-09-26

**Authors:** Olga Amelkina, Andreia M. da Silva, Alexandre R. Silva, Pierre Comizzoli

**Affiliations:** ^1^ Smithsonian’s National Zoo and Conservation Biology Institute, Washington, DC, United States; ^2^ Laboratory of Animal Germplasm Conservation, Federal Rural University of Semi-Arid—UFERSA, Mossoró, Brazil

**Keywords:** domestic cat (*Felis catus*), microRNA, ovary, testis, RNA-seq

## Abstract

The aim of the study was to perform the first in-depth analysis of miRNAs in ovarian and testicular tissues of the domestic cat, a critical biomedical model. Specifically, potential miRNA involvement was explored in gonadal function, testis development, and cellular stress response to preservation protocols. We performed miRNA-sequencing on 20 ovarian and 20 testicular samples from 15 cats, including different ages and tissue treatments. Using fresh tissues (*n* = 15), we confirmed gonadal expression of 183 miRNA precursors and discovered additional 52 novel feline candidate precursors. We integrated the mRNA data from our previous study on the same age and treatment groups to create in-silico miRNA-mRNA networks and their functional enrichment, which allows comprehensive exploration into possible miRNA functions in cat gonads. Clusters of miRNAs united by shared differentially expressed mRNA targets are potentially involved in testicular development and spermatogenesis. MicroRNAs could play a significant role in ovarian tissue response to stress from microwave-assisted dehydration, with smaller roles in cellular response to vitrification in both ovary and testis. This new list of miRNAs with potential function in cat gonads is a major step towards understanding the gonadal biology, as well as optimizing fertility preservation protocols.

## Introduction

The domestic cat is an invaluable model for developing protocols of fertility preservation and assisted reproduction in wild felids (Comizzoli et al., 2010; Comizzoli, 2015). It is also an important biomedical model, carrying a similarity with human genome with high degree of conserved synteny and serving as a model for ∼250 genetic diseases that are analogous to human disorders (O’Brien et al., 2002; Pontius et al., 2007; Tamazian et al., 2014). Recently, our group has been focusing on elucidating transcriptome dynamics in the ovarian and testicular tissue of domestic cat to better understand molecular mechanisms behind gonadal development and response of tissues to stress caused by preservation protocols (Amelkina and Comizzoli, 2020; Amelkina et al., 2021). We have reported major changes in mRNA expression in the ovarian tissue in response to vitrification and microwave-assisted preservation (Amelkina and Comizzoli, 2020), as well as gradual shifts in transcriptome dynamics throughout testicular development (Amelkina et al., 2021). To better understand the processes observed in these gonadal tissues, we need to go further and unravel the mechanisms of gene expression regulation.

One of the regulators of gene expression are non-coding miRNAs, and, specifically, microRNAs (miRNAs). These miRNAs (approximately 22 nucleotides in length) are highly conserved between species and have been estimated to modulate up to 60% of protein-coding genes in the human genome (Lewis et al., 2005). MicroRNAs have been shown to be involved in many physiological processes, including differentiation, proliferation and cell cycle progression, development, immune response, and apoptosis (Friedman et al., 2009). In gonadal tissues, miRNAs have been shown to participate in the regulation of spermatogenesis and folliculogenesis (Li et al., 2015; He et al., 2021; Walker, 2022). miRNAs are also known to regulate cellular stress responses by working together with transcription factors and protein partners, and being referred to as guardians of cellular homeostasis (Leung and Sharp, 2010; Mendell and Olson, 2012; Olejniczak et al., 2018). In addition, a family of miRNAs termed CryoMiRs have been shown to be associated with freezing tolerance, and regulate metabolism in mammalian hibernators and insects (Lyons et al., 2013; Singh and Storey, 2021). Finally, changes in miRNA expressions have been identified in cryopreserved oocytes, embryos and spermatozoa compared to fresh counterparts in human (Daneshvar et al., 2021), mouse (Heidari et al., 2019; Li et al., 2019; Movahed et al., 2019; Daneshvar et al., 2021; Xu et al., 2021), cow (Shangguan et al., 2020) and pig (Zhang et al., 2017; Dai et al., 2019).

Cellular biogenesis of miRNAs consists of several steps and starts with transcription of miRNA genes, mainly by RNA polymerase II, and generation of long primary transcripts (pri-miRNA) that are capped and polyadenylated (Krol et al., 2010; Ha and Kim, 2014). These pri-miRNA are then processes into hairpin structures of ∼60–80 nt known as miRNA precursors and transported to the cytoplasm, where they are further processed by sequential activity of the RNase III-type endonucleases Drosha and Dicer, resulting in the production of mature miRNAs of ∼21–22 nt in length (Kim et al., 2009). Binding of miRNA to the 3′UTR of target mRNAs is accomplished by incorporating one strand of mature miRNA into one of the four Argonaute proteins to form the RNA-induced silencing complex (RISC). Silencing of mRNA can then be done by translation inhibition, mRNA cleavage, deadenylation of the poly A tail and degradation, or translocation to P-body compartments for storage or degradation (Catalanotto et al., 2016).

Currently, there is no record for the domestic cat in the largest miRNA database, miRBase (Kozomara and Griffiths-Jones, 2014). Only one study previously used small-RNA sequencing to profile miRNAs in normal cat tissues, including two ovarian and two testicular samples of unknown developmental stage (Laganà et al., 2017). Additional studies include computational prediction of miRNAs within the cat genome (Pontius et al., 2007; Tamazian et al., 2014), miRNA profiling in feline kidney cell line (Sun et al., 2014), in liver following acute infection (Cong et al., 2017) and in serum (Fleischhacker et al., 2013; Weber et al., 2015). As miRNAs are known to have a high tissue- and stage-specific expression, it is important to profile several samples of tissues at a known developmental stage to obtain a full list of miRNAs for the species. Additionally, establishment of a miRNA expression profile in gonadal tissues of the domestic cat is an essential prerequisite for comprehensively exploring the biological function of miRNAs in feline reproduction. Analysis of changes in miRNA expression in the tissue depending on the condition, such as vitrified/thawed or dried/rehydrated, contributes to the understanding of miRNA function in cellular stress response, as well as in identification of miRNA biomarkers of cellular stress. Finally, integrating data on mRNA expression into miRNA analysis is essential for being a step closer to understanding the whole picture of the regulation of gene expression and associated functional changes in gonadal tissues.

The present study aimed to perform an in-depth analysis of miRNAs in ovarian and testicular tissues of the domestic cat, creating a comprehensive list of feline gonadal miRNAs and exploring potential miRNA involvement in three main processes: gonadal function, testis development, and cellular stress response. Specifically, objectives were to 1) identify known and novel miRNAs in the domestic cat ovarian and testicular tissue, 2) characterize tissue- and stage-specific expression of miRNAs in ovary and testis of prepubertal and/or adult cats and 3) using previously obtained mRNA data, build miRNA-mRNA interaction networks to study transcriptomic and functional changes in gonadal tissues in response to preservation protocols, as well as in testicular tissue throughout its development.

## Materials and methods

### Ethical statement

This study did not require the approval of the Animal Care and Use Committee of the Smithsonian Conservation Biology Institute because cat gonads were collected at local veterinary clinics as byproducts from owner-requested routine ovario-hysterectomies and orchiectomies.

### Experiment design, sample collection and tissue processing

**FIGURE 1:**
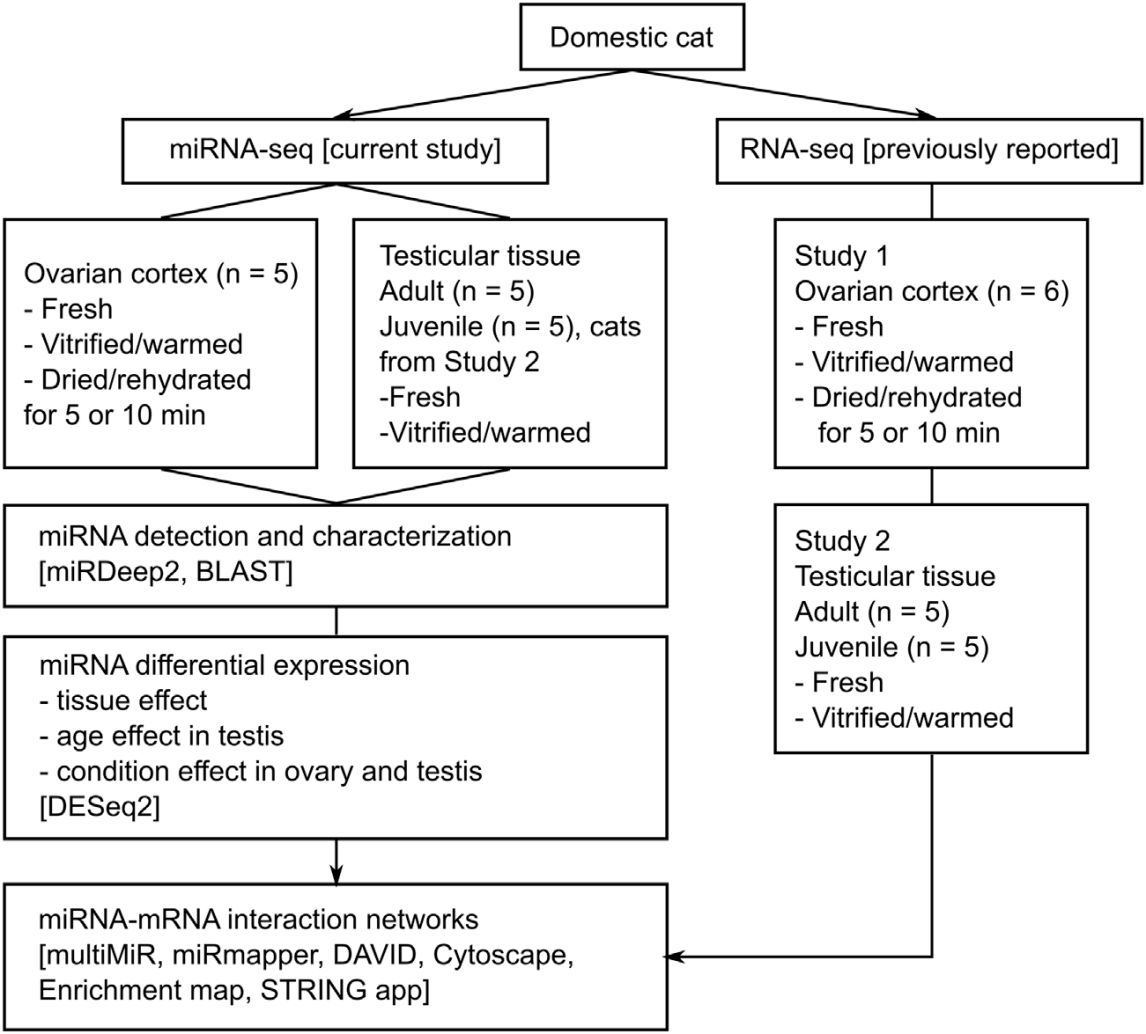
Experimental design. Study 1 And Study 2 refer to our previous reports on mRNA expression in domestic cat ovarian (Amelkina and Comizzoli, 2020) and testicular (Amelkina et al., 2021) tissues, respectively.

In the current study, we integrate previously obtained mRNA data on domestic cat (*Felis catus*) from Study 1, BioProject PRJNA662384, (Amelkina and Comizzoli, 2020), and Study 2, BioProject PRJNA741252, (Amelkina et al., 2021), for miRNA-mRNA analysis (Figure 1). All conditions and protocols used for tissue processing and experiments were the same as in Study 1 (Amelkina and Comizzoli, 2020) and 2 (Amelkina et al., 2021). Tissue collection and dissection, as well as vitrification (Mouttham and Comizzoli, 2016; Amelkina and Comizzoli, 2020) or dehydration (Lee et al., 2019; Amelkina and Comizzoli, 2020) of ovarian cortex, and vitrification of testicular tissues (Lima et al., 2018; Amelkina et al., 2021) were performed using protocols reported previously for domestic cat (Supplementary Methods online). Three out of five cats that were used for the ovarian cortex analysis were the same as the ones used for RNA-seq in Study 1 (Amelkina and Comizzoli, 2020). Samples that were used for the testicular tissue analysis were the same as in Study 2 (Amelkina et al., 2021), except for two adult cats. Supplementary Table S1.1 contains detailed information about samples, including their corresponding RNA-seq IDs from Studies 1 and 2.

### RNA isolation, library preparation and miRNA sequencing

Total RNA was isolated from up to 10 mg of tissue using mirVana™ miRNA Isolation Kit (Life Technologies); tissue was homogenized in RNA lysis buffer using TissueLyser (Qiagen, Hilden, Germany; 2 × 2 min at 30 Hz; 5 mm stainless steel beads).

Concentration, purity, and integrity of isolated RNA was measured with Qubit™ RNA HS Assay Kit (Invitrogen), NanoDrop™ spectrophotometer (Thermo Fisher Scientific) and TapeStation RNA HS Kit (Agilent Technologies, Santa Clara, CA), respectively. Purified RNA was stored in nuclease-free water at -80°C until library preparation. Isolated RNA was sent to RealSeq Biosciences core to prepare a total of 40 libraries using the RealSeq®-AC method of preparing miRNA sequencing libraries that involves ligating the miRNAs with a single adapter and circulazing the ligation products (Barberán-Soler et al., 2018). When compared to other methods, RealSeq®-AC provides greatly reduces miRNA sequencing bias and allows the identification of the largest variety of miRNAs in biological samples (Barberán-Soler et al., 2018). Libraries were amplified by PCR and sequenced in two NextSeq 550 runs with the NextSeq 500/550 High Output Kit v2.5 (75 cycles). Sequencing was done with single end 75 nt reads and dual 6 nt indexes. Libraries were loaded at 1.8 p.m. and sequenced with RealSeq Biosciences custom sequencing primer for read one; 15% PhiX control was used.

### Data pre-processing

Here and below, all data manipulations were performed using the Smithsonian Institution High Performance Computing Cluster (doi.org/10.25572/SIHPC). Raw sequencing files were merged for each sample to generate a single fastq file per sample. The quality of produced data was determined by the phred quality score at each cycle using FastQC (v. 0.11.8). Cutadapt 2.3 (Martin, 2011) was used to remove adapter sequences and all reads with inserts smaller than 15 nt applying the following parameters: --nextseq-trim = 15 -u 1 -a TGG​AAT​TCT​CGG​GTG​CCA​AGG -m 15. The “-u 1” was used to remove the first base of each read necessary for RealSeq method. Trimmed reads were aligned to the cat genome (GCF_000181335.3_Felis_catus_9.0) using short read aligner Bowtie (Langmead et al., 2009). Reads and trimming statistics can be found in Supplementary Table S1.1.

### Identification of known and novel miRNAs

The miRNA identification analysis was performed using miRDeep2 (v. 2.0.1.2) (Mackowiak, 2011) independently in each fresh ovarian (*n* = 5) and testicular (adult, *n* = 5; juvenile, *n* = 5) sample to allow for authentic detection of miRNAs. Previously reported domestic cat precursors and mature miRNAs (Laganà et al., 2017), as well as human and dog mature miRNAs from miRBase (v22.1), were used as additional input when running miRDeep2.pl module. MiRDeep2 algorithm excises the sRNA sequence and computes their secondary RNA structure with RNAfold (Gruber et al., 2008) to predict miRNA precursors that are later scored for their likelihood (Friedländer et al., 2008). To prevent false positive detection of miRNA stem-loops, the signal-to-noise ratios estimated over 100 rounds of independent permutations were calculated for different miRDeep log-odds score cut-offs from -10 to 10. Each output per sample was filtered for: 1) miRDeep2 score above 4, 2) no Rfam alert (Griffiths-Jones et al., 2005), 3) significant RNAfold value and 4) number of mature reads above 10. Identified miRNAs that were not reported in the previous domestic cat study (Laganà et al., 2017) were considered putative novel feline miRNAs. Each novel miRNA was assigned random ID and extracted mature and hairpin sequences were further used for BLAST analysis utilizing miRBase database (v22.1). For identification of mature and star miRNAs by BLAST, the following options were used: −strand plus -evalue 10 -word_size 4 -penalty −4 -reward 5 -gapopen 8 -gapextend 6. For identification of precursor miRNAs, the same parameters were used, except the minimum e_value was set to 0.001 and hits were filtered for >80% identity and >80% overlap using awk. Each output was checked, and best representative hits were selected using the following criteria: lowest e-value, highest coverage, expressed in highest number of samples. MiRNAs that differed only by additional two nucleotides at the start/end position were considered as a single miRNA and the miRNA that was identified in the highest number of samples or had a higher miRDeep2 score was chosen as a representative and most likely true miRNA.

Putative precursors that did not return BLAST hit were further filtered with the following criteria: 1) expression in at least three samples, 2) no hits for hairpin sequence in databases of RefSeq, rRNA, 16S, 18S, 28S; 3) no repetitive sequences (complexity). Filtered hairpins were then aligned against cat genome and any putative precursor that provided significant BLAST hits, defined as e-value  ≤ 1× e-6 against multiple loci (>5) in the domestic cat genome reference sequence, were considered to be part of interspersed repeats or tandem repeats and, consequently, excluded as putative novel miRNAs. Remaining unknown hairpins were submitted to rtools for secondary structure prediction and validation (http://rtools.cbrc.jp/).

### miRNA quantification

Known and novel feline miRNAs were combined and the expression for each sample was determined using miRDeep2 quantifier module. Mature miRNAs expressed at < 1 cpm in 90% of samples were filtered out. Then, miRNAs expressed at ≥ 10 cpm in 60% of fresh samples (3 out of 5 samples in a group) were considered as expressed in the tissue analyzed. miRDeep2 normalization (cpm) normalizes expression values by library size and multiplies them by a factor of 1E6 which corresponds technically to counts per million mapped miRNA reads. The library size is the total number of reads mapping to miRNA precursors. To analyze whether miRNAs were ubiquitously expressed among the targeted tissues, a Venn diagram was plotted by using VennDiagram R package.

### Differential expression and gene set analysis

Differential expression analysis was performed on data obtained from 40 samples using the DESeq2 R package (Love et al., 2014). For visualization, size factors were estimated from the count data and the relative log expression (RLE) normalization was used to obtain regularized log transformed values. These normalized values were then used for principal component analysis (plotPCA function in DESeq2 R package) and creation of clustered heatmaps (pheatmap R package). Wald test was used on genes that passed an independent filtering step and resulting *p* values were adjusted for multiple testing using the Benjamini–Hochberg procedure. The following groups were formed for differential expression analysis: fresh ovarian tissues (OvF, *n* = 5), fresh testicular tissues (TesF, *n* = 10, includes 5 adults, Tes.AF, and 5 juveniles, Tes.JF), ovarian tissues dehydrated for 5 min (Ov.D5, *n* = 5), for 10 min (Ov.D10, *n* = 5) or vitrified (Ov.V, *n* = 5), vitrified testicular tissues in adults (Tes.AV, *n* = 5) and juveniles (Tes.JV, *n* = 5). For tissue effect, Ov.F was compared with TesF group; for age effect, Tes.AF was compared with Tes.JF group; for condition effect in ovary, Ov.D5, Ov.D10, Ov.V and Ov.F were all used for pairwise comparison between each other; for condition effect in testis, Tes.AV and Tes.JV were compared to Tes.AF and Tes.JF, respectively. *p*-value and miRNA expression level were used as filtering conditions for each effect (details for each miRNA are available in Supplementary Tables S1–3).

For mature miRNAs differentially expressed in fresh ovary vs. testis, gene set analysis was performed using RBiomirGS R Package (Zhang and Storey, 2018). This package uses fold change and *p* value of differentially expressed miRNAs to calculate a miRNA expression score for each miRNA measured. A miRNA impact score is then generated for target mRNAs, and gene set enrichment is conducted using logistic regression (Zhang and Storey, 2018).

### *In silico* miRNA-mRNA networks

Data on differentially expressed mRNAs was obtained from previously reported Study 1 (Amelkina and Comizzoli, 2020) (ovarian tissue) and Study 2 (Amelkina et al., 2021) (testicular tissue). For each DE mRNA, human orthologs were identified using orthologsBioMART R Package; human orthologs for DE miRNAs were identified using BLAST at miRNA discovery step. Correlation of miRNA-gene targets for upregulated and downregulated DE mRNAs were acquired using multiMiR (Ru et al., 2014) R Package, considering only the top 35% of predicted interactions. Networks of predicted DE miRNA-mRNA interactions were then built using Cytoscape software (v. 3.8.2) (Cline et al., 2007). Finally, miRmapper (da Silveira et al., 2018) R Package was used on the obtained list of miRNA-target gene interactions and a list of all DE mRNAs to build adjacency matrices for calculation of miRNA similarity utilizing Jaccard distance, and to measure the miRNA impact.

### Functional enrichment analysis of differentially expressed mRNA targets

Differentially expressed mRNA targets were used for gene-set functional enrichment analysis with DAVID tool (Huang et al., 2009), setting species to domestic cat. For each comparison pair, total number of targets and separately up- and downregulated targets were analyzed. EASE score (modified Fisher Exact *p*-value of enrichment) was set to 0.05. Functional enrichment networks were built based on DAVID output charts of gene-set enrichment for each DE miRNA and its DE targets using Enrichment Map app (v. 3.3.3) (Merico et al., 2010) in Cytoscape with Overlap set to 0.5. Autoannotate App (v. 1.3.4) with MLC algorithm based on similarity coefficient was used to create annotated groups, which were then manually adjusted based on biological meaning.

### *In silico* protein-protein interaction analysis using STRING

In silico protein-protein interaction analysis of differentially expressed mRNA targets was performed on the basis of the STRING database for the domestic cat (Szklarczyk et al., 2017). Interaction networks were built based on the list of mRNA targets for each comparison pair using stringApp (v. 1.7.0) (Doncheva et al., 2019) in Cytoscape with confidence cutoff score set to 0.4, and then integrated with the miRNA-mRNA networks. Functional enrichment of formed clusters was performed using domestic cat genome as a background, enriched terms were analyzed with varying redundancy cutoff settings.

## Results

### Known and novel miRNAs identified in domestic cat gonads

To define and characterize the cat gonadal miRNAome, we generated and sequenced 40 small-RNA libraries from ovarian and testicular tissue samples of 15 cats (Figure 1; Supplementary Table S1.1). Out of these, 15 samples representing fresh gonadal tissues were used for miRNA detection using miRDeep2, a computational tool to map, analyze and score deep sequencing data for the identification of known and novel miRNAs (An et al., 2013).

Previous report on domestic cat miRNAome identified 271 candidate miRNA precursors, encoding a total of 475 mature sequences (Laganà et al., 2017). Out of these previously reported miRNAs, we identified 183 miRNA precursors with high enough score and detectable expression for at least one arm. Two of these miRNAs precursors didn’t have orthologs and were identified by Laganà et al. as fca-mir-chrE3_33626 and fca-mir-chrX_338640 (Laganà et al., 2017). We additionally identified 52 candidate miRNA precursors encoding a total of 92 mature sequences that were previously not reported in domestic cat and were thus considered novel. We analyzed conservation, tissue expression, genomic location, and arm preference for all identified miRNAs. The results of this analysis are summarized in Supplementary Table S1.2–3 online.

Additionally, we identified one putative miRNA precursor on X chromosome encoding both 3p and 5p mature sequences that was not reported in any species previously, temporarily termed fca-mir-chrX_QO3CD. The analysis of secondary structure of this miRNA using generalized centroid estimators (Hamada et al., 2009) showed that it forms a hairpin with high probability and is a good candidate for novel miRNA (Supplementary Figure S1 online).

### Distribution of miRNAs across gonadal tissues

**FIGURE 2:**
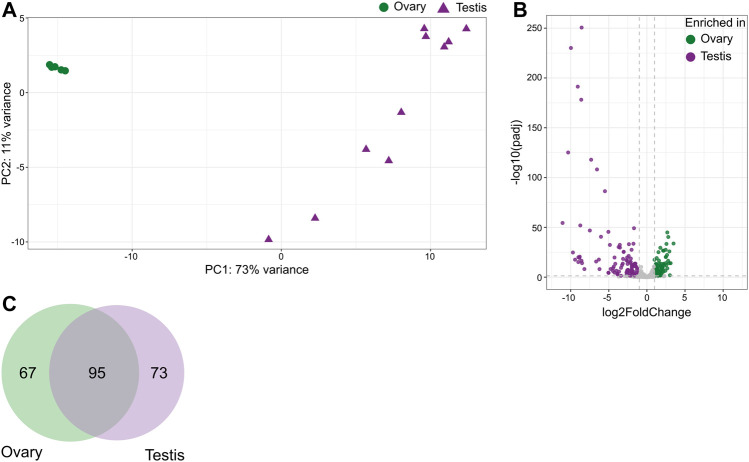
Distribution of miRNAs across gonadal tissues in the domestic cat. **(A)** Principal component analysis plot representing variations of mature miRNAs in ovarian and testicular samples. **(B)** Volcano plot showing differentially expressed mature miRNAs in ovary vs. testis tissues (Wald test, adjusted *p*-value < 0.05, absolute fold change ≥2). **(C)** Venn diagram of miRNAs precursors enriched in either ovary or testis.

Distribution of miRNAs based on their overall expression was clearly divided into two groups representing ovary and testis (Figure 2A). Differential expression analysis between fresh tissue samples from ovary vs. testis returned 94 mature miRNAs enriched in ovary and 106 in testis, with 227 enriched in both tissues (Figure 2B; Supplementary Table S1.4). These corresponded to 67 precursor miRNAs enriched in ovary and 73 in testis, while 95 precursor miRNAs were similarly expressed in both gonadal tissues (Figure 2C; Supplementary Table S1.2). For novel miRNA fca-mir-chrX_QO3CD, both arms were enriched in testicular tissues. Out of all our identified miRNAs, the following precursors were also previously reported to be enriched in feline ovary and/or testis: 1) fca-mir-135b reported in testis, 2) fca-mir-34c, fca-mir-424, fca-mir-449 and fca-mir-503 reported in ovary, 3) fca-mir-202, fca-mir-506, fca-mir-508, fca-mir-514, and fca-mir-chrX_38640 reported in both gonads (Laganà et al., 2017).

Gene set analysis of mature miRNAs enriched in either ovary or testis returned over 200 functions and pathways from Gene Ontology and KEGG databases. Top 50 enriched gene sets based on validated human target mRNAs are presented in Supplementary Figure S2. Pathways and functions enriched in the ovary included various signaling pathways, oocyte maturation and meiosis, while steroid hormone biosynthesis, olfactory transduction, sperm motility and axoneme assembly were enriched in testis.

### Clusters of miRNAs are potentially involved in testicular development and spermatogenesis

**FIGURE 3:**
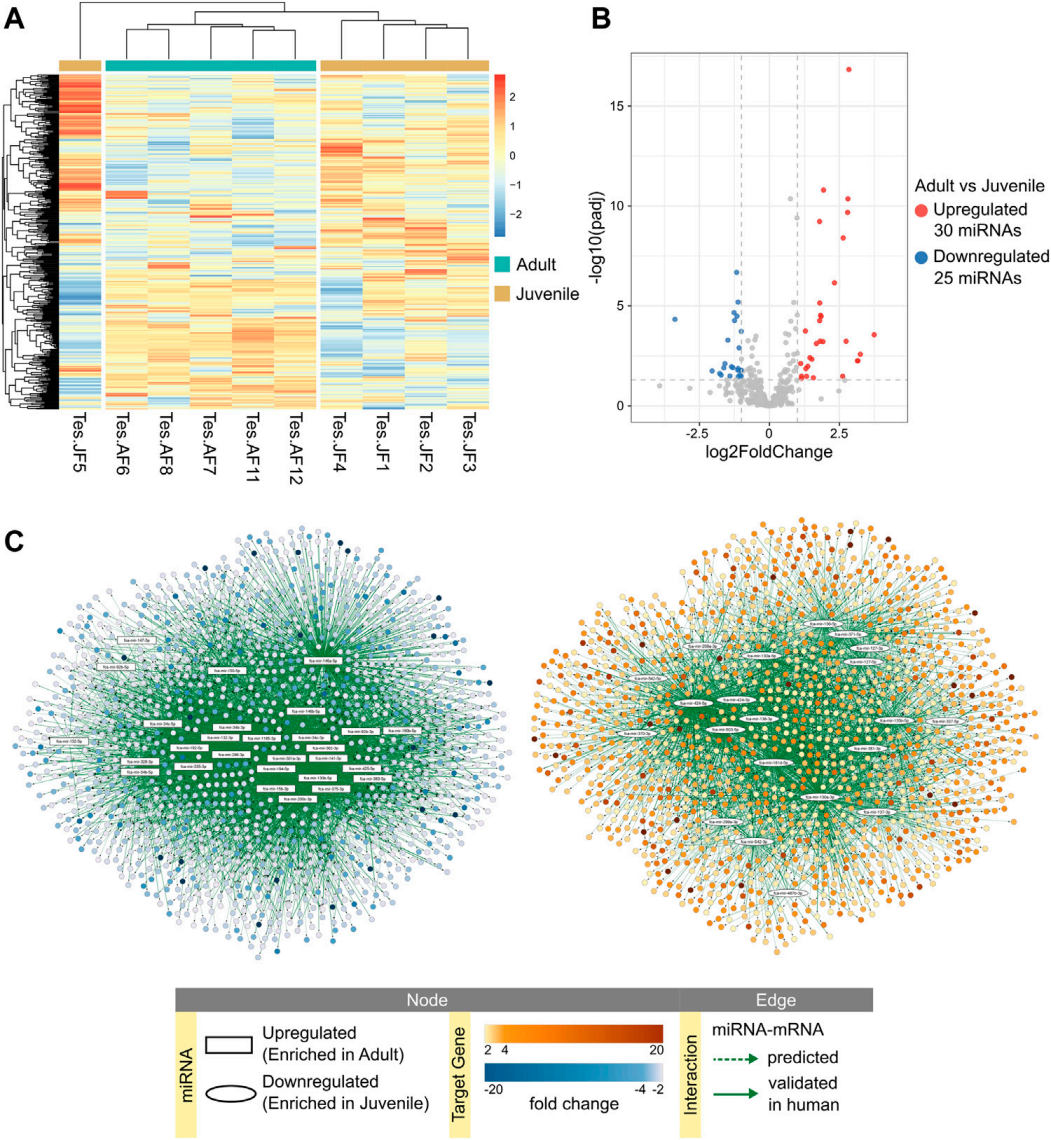
miRNA expression in adult and juvenile testicular tissues. **(A)** Heatmap of one-way hierarchical clustering analysis (Euclidian method, complete linkage) using Z-score for RLE normalized values of all mature miRNAs identified in adult and juvenile testes. **(B)** Volcano plot showing differentially expressed mature miRNAs in adult vs. juvenile testes (Wald test, adjusted *p*-value < 0.05, absolute fold change ≥2). **(C)** miRNA-mRNA predicted interaction networks enriched in adult or juvenile testes. Full size interactive networks with corresponding data tables are available in Supplementary Data Sheet S1.

The expression of mature miRNAs was clearly divided between testicular tissues obtained from adult and juvenile cats (Figure 3A). Differential expression analysis revealed 30 mature miRNAs enriched in adult and 25 in juvenile testis (Figure 3B; Supplementary Table S2.1). We used our previous data on mRNA expression in cat testis (Amelkina et al., 2021) for in-silico analysis to create miRNA-mRNA networks that represent miRNA regulation of mRNAs throughout testis development from juvenile to adult age (Figure 3C; Supplementary Table S2.2–3). Because we used the same juvenile individuals as in our previous RNA-seq study (Amelkina et al., 2021), we could divide the juvenile group further into early and late juvenile. We performed differential expression analysis and in-silico analysis of miRNA-mRNA interactions for comparison groups of adult vs. early juvenile, adult vs. late juvenile and late vs. early juvenile (Supplementary Figure S2; Supplementary Table S2). We then used the detected differentially expressed target mRNAs for each differentially expressed miRNA for functional enrichment. Full size interactive miRNA-mRNA network with corresponding data table is available in Supplementary Data Sheet S1.

**FIGURE 4:**
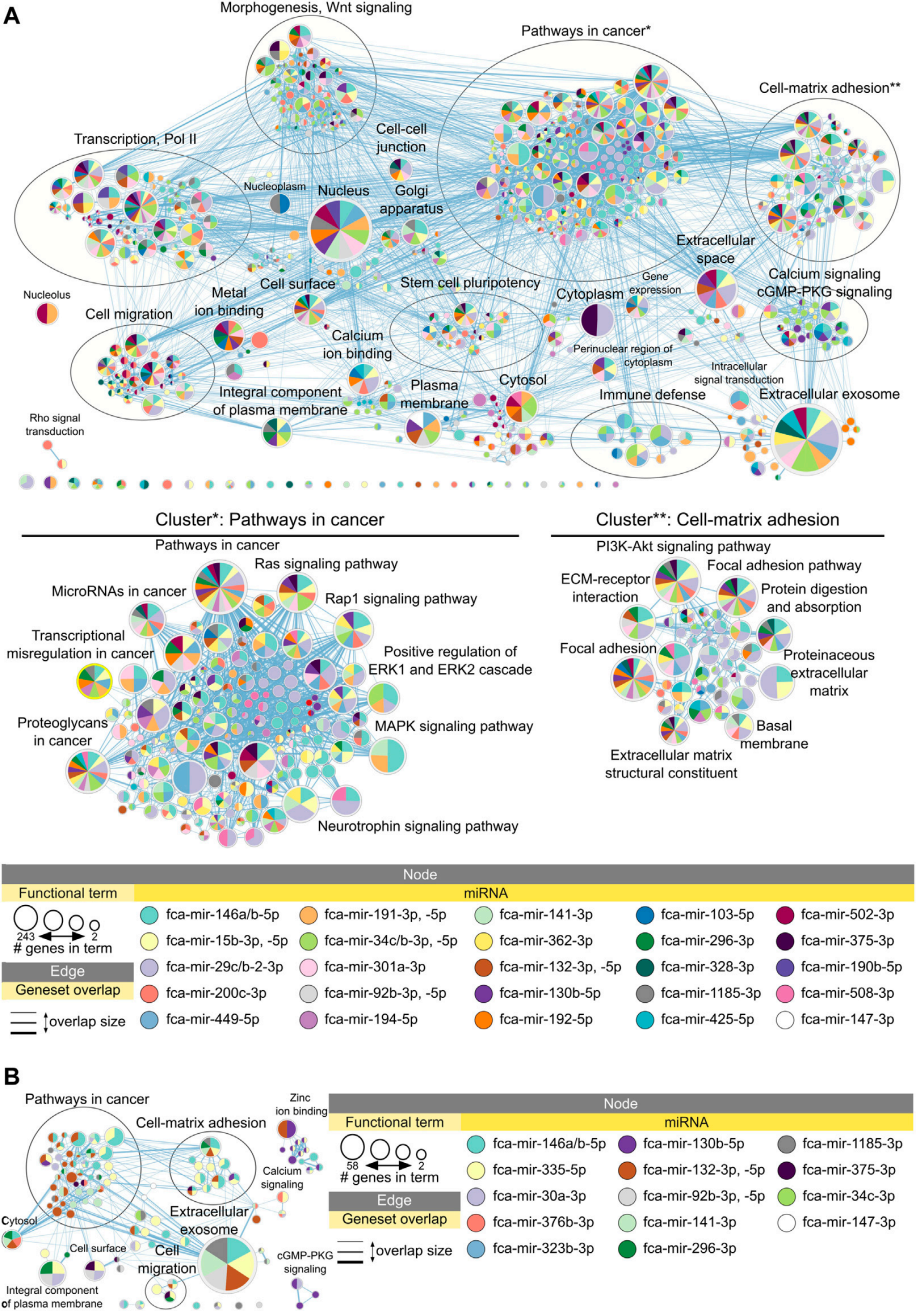
Functional terms and pathways potentially inhibited by miRNAs in adult testes. Enrichment map displays enriched gene-sets based on downregulated mRNA targets for each upregulated miRNA in **(A)** adult vs. early juvenile testis, i.e., enriched in earl juvenile, and **(B)** adult vs. late juvenile testis, i.e., enriched in late juvenile. Interactive networks including networks of each functional cluster with corresponding data tables are available in Supplementary Data Sheet S2.

Figure 4A shows enrichment map of functional terms potentially downregulated by upregulated miRNAs in adult compared to early juvenile testicular tissues, i.e., functions enriched in early juvenile testis (Supplementary Data Sheet S2 for interactive view). Several clusters can be identified representing processes of cell-matrix adhesion, morphogenesis, stem cell pluripotency, cell migration and immune defense (Figure 4A). The biggest functional cluster inhibited in adult tissues by miRNAs unites terms related to pathways in cancer, including Ras/Rap1/MAPK signaling pathways. Another big cluster inhibited in adult tissues compared to early juvenile is related to cell-matrix adhesion and includes PI3K-Akt signaling pathway. As seen in Figure 4A, no single miRNA is responsible for potential inhibition of functional cluster, but rather a cluster of miRNAs is responsible for regulation of each functional process. Supplementary Figure S3 shows that miRNAs form actual clusters based on the similarity of their differentially expressed targets. Table 1 lists all miRNAs enriched in adult tissues with a summary of their differential expression, impact on differentially expressed mRNAs and involvement in each functional cluster. When comparing adult to late juvenile, miRNAs show predicted inhibition of cell-matrix adhesion, pathways in cancer and cell migration (Figure 4B; Table 1), which excludes clusters of morphogenesis, stem cell pluripotency and immune defense enriched in early juvenile.

**TABLE 1:** Impact of upregulated miRNAs on downregulated mRNA in Adult compared to Early and Late Juvenile testis.

miRNA	miRNA family	Upregulated compared to	Base mean	Fold change	Adjusted *p*-value	DE targets	Targets, %	DEGs, %	Functional cluster of DE targets
fca-mir-146a-5p	mir-146	Early and Late Juvenile	3,207	3.7	1.52E-08	658	36.2	27.8	PC, C-MA, M-Wnt, SCP, IR, CM, TP, ST
fca-mir-146b-5p			833	4.5	3.63E-13	146	8.0	6.2	
fca-mir-15b-3p	mir-15	Early Juvenile	21	4.2	0.0005	78	4.3	3.3	
fca-mir-15b-5p			1,409	2.2	1.03E-05	475	26.1	20.1	
fca-mir-29b-2-3p	mir-29	Early Juvenile	1,478	2.3	0.0006	347	19.1	14.6	
fca-mir-29c-3p			10,225	2.9	0.0004	441	24.2	18.6	
fca-mir-449-3p	mir-449	Early Juvenile	76	16.3	0.0005	-	-	14.3	
fca-mir-449-5p			7,673	19.6	0.0016	339	18.6	14.3	
fca-mir-34c-3p*	mir-34	Early and Late Juvenile	649	19.8	3.45E-13	58	3.2	2.4	
fca-mir-34c-5p		Early Juvenile	46,247	23.9	1.71E-06	280	15.4	11.8	
fca-mir-34b-3p			541	22.8	3.32E-05	89	4.9	3.8	
fca-mir-191-3p	mir-191	Early Juvenile	47	2.3	0.0029	18	1.0	0.8	PC, C-MA, M-Wnt, SCP, IR, CM, TP
fca-mir-191-5p			77,269	2.3	2.30E-07	336	18.5	14.2	
fca-mir-92b-3p	mir-25	Early and Late Juvenile	35	5.2	2.97E-07	273	15.0	11.5	
fca-mir-92b-5p			18	5.2	1.20E-05	24	1.3	1.0	
fca-mir-200c-3p*	mir-8	Early Juvenile	44	5.0	5.36E-06	366	20.1	15.4	PC, C-MA, M-Wnt, SCP, CM, TP
fca-mir-141-3p		Early and Late Juvenile	284	4.1	2.14E-06	231	12.7	9.8	PC, C-MA, M-Wnt, CM, TP
fca-mir-130b-5p	mir-130	Early and Late Juvenile	17	3.7	0.0008	187	10.3	7.9	PC, C-MA, M-Wnt, CM, TP, ST
fca-mir-301a-3p		Early Juvenile	369	3.6	1.20E-05	277	15.2	11.7	PC, C-MA, M-Wnt, SCP, CM, TP
fca-mir-132-3p	mir-132	Early and Late Juvenile	821	7.6	1.75E-14	203	11.2	8.6	PC, C-MA, M-Wnt, SCP, CM, TP, ST
fca-mir-132-5p			31	5.6	2.54E-07	29	1.6	1.2	
fca-mir-375-3p	mir-375		930	49.4	8.94E-22	117	6.4	4.9	
fca-mir-362-3p	mir-362	Early Juvenile	158	2.9	1.45E-05	207	11.4	8.7	PC, C-MA, M-Wnt, SCP, CM, TP
fca-mir-192-5p	mir-192		32	5.5	5.05E-06	172	9.5	7.3	
fca-mir-296-3p	mir-296	Early and Late Juvenile	111	5.3	3.73E-09	164	9.0	6.9	
fca-mir-296-5p			28	8.2	9.74E-09	-	-	-	
fca-mir-1185-3p	mir-154	Early and Late Juvenile	37	3.0	0.0072	148	8.1	6.2	
fca-mir-323b-3p^		Late Juvenile	37	2.4	0.0330	8	2.4	1.6	ST
fca-mir-194-5p	mir-194	Early Juvenile	52	4.6	0.0003	234	12.9	9.9	PC, C-MA, M-Wnt, CM, TP, ST
fca-mir-103-5p	mir-103		15	2.8	0.0243	164	9.0	6.9	PC, C-MA, SCP, CM, TP, ST
fca-mir-502-3p	mir-500		578	2.1	0.0028	135	7.4	5.7	C-MA, M-Wnt, CM, TP, ST
fca-mir-425-5p	mir-425		1,295	3.1	3.08E-07	135	7.4	5.7	C-MA, M-Wnt, CM, ST
fca-mir-328-3p	mir-328		10	3.5	0.0086	135	7.4	5.7	PC, C-MA, CM
fca-mir-190b-5p	mir-190		85	3.0	0.0039	66	3.6	2.8	TP
fca-mir-508-3p	mir-506		5,408	2.4	0.0035	60	3.3	2.5	PC, C-MA, TP
fca-mir-508-5p			526	2.2	0.0018	-	-	-	
fca-mir-506-5p			1,628	2.2	0.0042	-	-	-	
fca-mir-335-3p^	mir-335	Late Juvenile	10	3.9	0.0417	148	45.3	29.8	PC, C-MA, ST
fca-mir-30a-3p^	mir-30		765	2.1	4.41E-05	50	15.3	10.1	PC, ST
fca-mir-376b-3p^	mir-368		179	2.7	0.0172	23	7.0	4.6	PC, ST
fca-mir-147-3p	mir-147	Early and Late Juvenile	56	9.7	1.24E-11	26	1.4	1.1	**-**

Data presented for comparison pair of Adult vs. Early Juvenile; if ^ - data from comparison pair Adult vs. Late Juvenile; * - also upregulated in Late vs. Early Juvenile. Targets, % - percentage of genes regulated by the miRNA out of all targets in the dataset. DEGs, % - percentage of genes impacted by miRNA out of all downregulated genes. DE, differentially expressed (downregulated). Functional clusters: C-MA, Cell-matrix adhesion; CM, Cell migration; IR, Immune response; M-Wnt, Morphogenesis, Wnt signaling; PC, Pathways in cancer; SCP, Stem cell pluripotency; ST, Signal transduction; TP, Transcription, Polymerase II.

**FIGURE 5:**
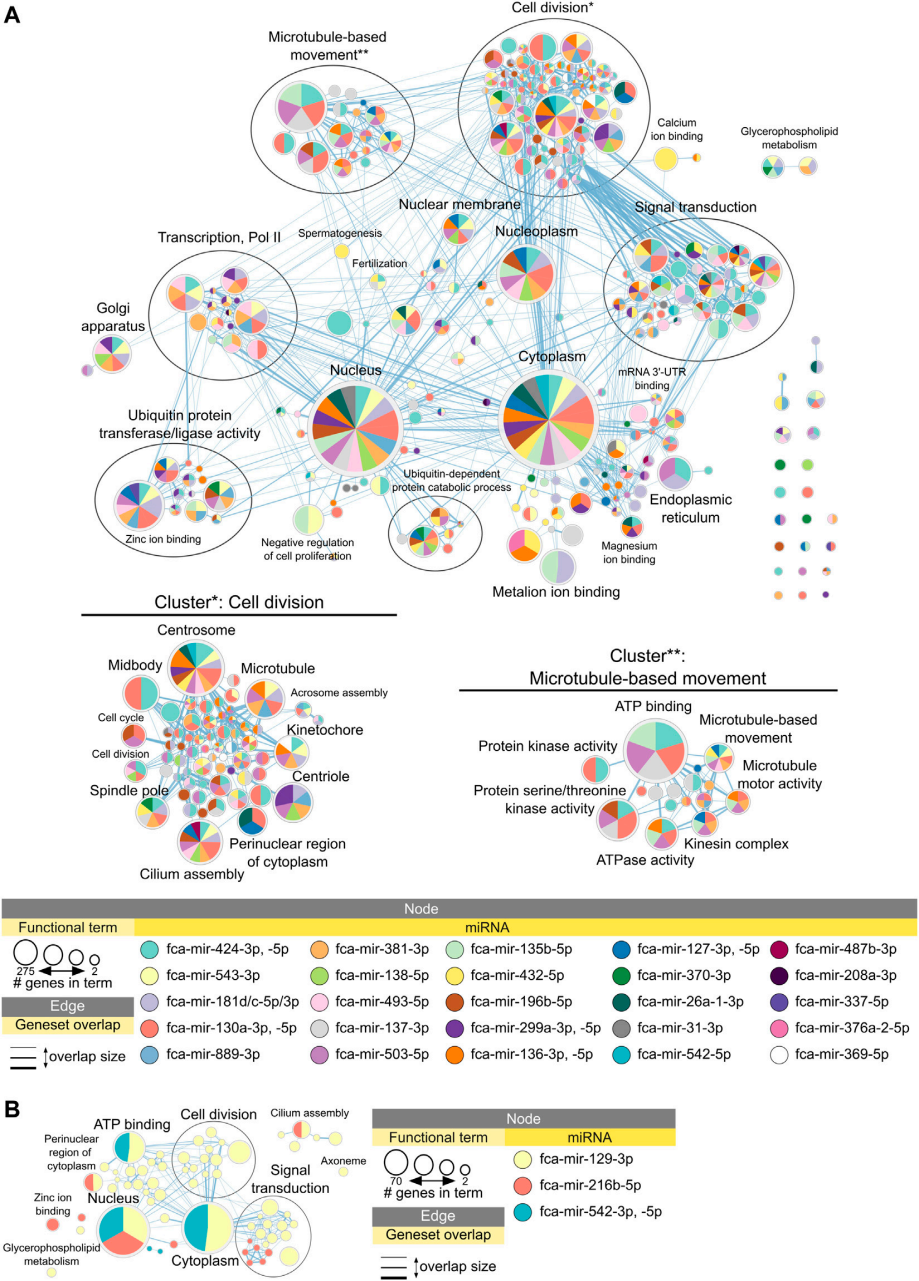
Functional terms and pathways potentially inhibited by miRNAs in juvenile testes. Enrichment map displays enriched gene-sets based on upregulated mRNA targets for each downregulated miRNA in **(A)** adult vs. early juvenile testis, i.e., enriched in adult compared to early juvenile, and **(B)** adult vs. late juvenile testis, i.e., enriched in adult compared to late juvenile. Interactive networks including networks of each functional cluster with corresponding data tables are available in Supplementary Data Sheet S2.

Figure 5A shows enrichment map with functional terms potentially downregulated by upregulated miRNAs in early juvenile compared to adult testicular tissues, i.e., functions enriched in adult testis (Supplementary Archive S2 for interactive view). Several clusters could be identified representing processes of cell division, microtubule-based movement, signal transduction and ubiquitin-related activity (Figure 5A). Figure 5B shows functional terms downregulated in late compared to adult testis, which include processes of cell division and signal transduction only. Table 2 summarized all miRNAs downregulated in adult compared to early and late juvenile testis with their calculated impact on differentially expressed mRNAs and related functional clusters.

**TABLE 2:** Impact of downregulated miRNAs on upregulated mRNA in Adult compared to Early and Late Juvenile testis.

miRNA	miRNA family	Downregulated compared to	Base mean	Fold change	Adjusted *p*-value	DE targets	Targets, %	DEGs, %	Functional cluster of DE targets
fca-mir-424-3p	mir-322	Early Juvenile	1,359	−2.5	1.39E-07	52	2.7	1.4	CD, M-BM, TP, Ub1, Ub2, ST
fca-mir-424-5p			13,804	−2.5	9.42E-10	520	26.5	14.1	
fca-mir-543-3p	mir-329		10	−3.7	0.01981	467	23.8	12.7	
fca-mir-130a-3p	mir-130		11,411	−2.8	0.0001	389	19.8	10.6	
fca-mir-130a-5p			15	−4.5	0.0033	336	17.1	9.1	
fca-mir-381-3p	mir-154		58	−3.1	0.0012	300	15.3	8.2	
fca-mir-181a-2-3p	mir-181		21	−2.5	0.0224	-	-	-	
fca-mir-181c-3p			45	−2.1	0.0033	38	1.9	1.0	CD, TP, Ub1, ST
fca-mir-181d-5p			295	−2.6	2.69E-07	405	20.7	11.0	
fca-mir-889-3p	mir-889		55	−2.4	0.0058	303	15.5	8.2	
fca-mir-299a-5p	mir-299		7	−3.7	0.0235	171	8.7	4.6	
fca-mir-299a-3p			34	−3.4	2.11E-05	80	4.1	2.2	
fca-mir-493-5p	mir-493		7	−3.8	0.0266	288	14.7	7.8	CD, M-BM, TP, Ub1, ST
fca-mir-135b-5p*	mir-135		31	−15.5	9.39E-09	241	12.3	6.5	M-BM, TP, Ub1, Ub2, ST
fca-mir-136-3p	mir-136		94	−2.6	0.0011	61	3.1	1.7	CD, M-BM, Ub1, ST
fca-mir-136-5p			177	−2.5	0.0025	157	8.0	4.3	
fca-mir-137-3p	mir-137		26	−3.4	0.0082	281	14.3	7.6	
fca-mir-138-5p	mir-138		928	−2.1	0.0137	299	15.3	8.1	CD, Ub2, ST
fca-mir-503-3p	mir-503		263	−2.4	3.71E-05	-	-	-	CD, M-BM, Ub1, Ub2, ST
fca-mir-503-5p			1,070	−2.8	5.52E-08	259	13.2	7.0	
fca-mir-432-5p	mir-432		66	−2.2	0.0236	224	11.4	6.1	CD, M-BM, TP, Ub2, ST
fca-mir-196b-5p	mir-196		128	−2.9	0.0301	206	10.5	5.6	CD, M-BM, Ub1, Ub2, ST
fca-mir-127-3p*	mir-127		234	−3.4	1.20E-05	31	1.6	0.8	
fca-mir-127-5p			8	−4.5	0.0062	148	7.6	4.0	
fca-mir-370-3p	mir-370		21	−3.5	0.0056	134	6.8	3.6	CD, Ub1, Ub2, ST
fca-mir-26a-1-3p	mir-26		8	−2.7	0.0406	78	4.0	2.1	CD, ST
fca-mir-542-3p^	mir-542	Late Juvenile	550	−2.2	0.0027	76	17.5	4.1	M-BM
fca-mir-542-5p		Early and Late Juvenile	3,256	−2.1	0.0018	28	1.4	0.8	ST
fca-mir-129-3p^	mir-129	Late Juvenile	1,463	−2.3	0.0069	345	79.5	18.8	M-BM, CD, ST
fca-mir-216b-5p^	mir-216		329	−4.2	0.0017	77	17.7	4.2	ST
fca-mir-208a-3p	mir-208	Early Juvenile	44	−2.3	0.0125	18	0.9	0.5	ST
fca-mir-337-5p	mir-337		29	−4.4	1.20E-05	15	0.8	0.4	Ub1, ST
fca-mir-31-3p	mir-31		27	−2.0	0.0212	48	2.4	1.3	CD
fca-mir-369-5p*	mir-154		186	−2.3	0.0012	9	0.5	0.2	
fca-mir-376a-2-5p*	mir-368		68	−2.8	0.0008	12	0.6	0.3	
fca-mir-376b-5p			8	−3.3	0.0084	6	0.3	0.2	
fca-mir-487b-3p	mir-154		57	−2.4	0.0018	19	1.0	0.5	
fca-mir-3959-5p	mir-379		235	−2.8	0.0003	-	-	-	-

Data presented for comparison pair of Adult vs. Early Juvenile; if ^ - data from comparison pair Adult vs. Late Juvenile; * - also downregulated in Late vs. Early Juvenile. Targets, % - percentage of genes regulated by the miRNA out of all targets in the dataset. DEGs, % - percentage of genes impacted by miRNA out of all downregulated genes. DE, differentially expressed (upregulated). Functional clusters: CD, cell division; M-BM –microtubule-based movement; TP, Transcription, Polymerase II; Ub1, Ubiquitin protein transferase/ligase activity; Ub2, Ubiquitin-dependent protein catabolic process; ST, Signal transduction.

Overall, miRNAs are apparently vastly involved in both processes of testis development and spermatogenesis in cat. Several miRNAs form a cluster and downregulate the same mRNA targets and functional clusters (Tables 1, 2).

### miRNAs are potentially involved in cellular stress response to preservation protocols

**FIGURE 6:**
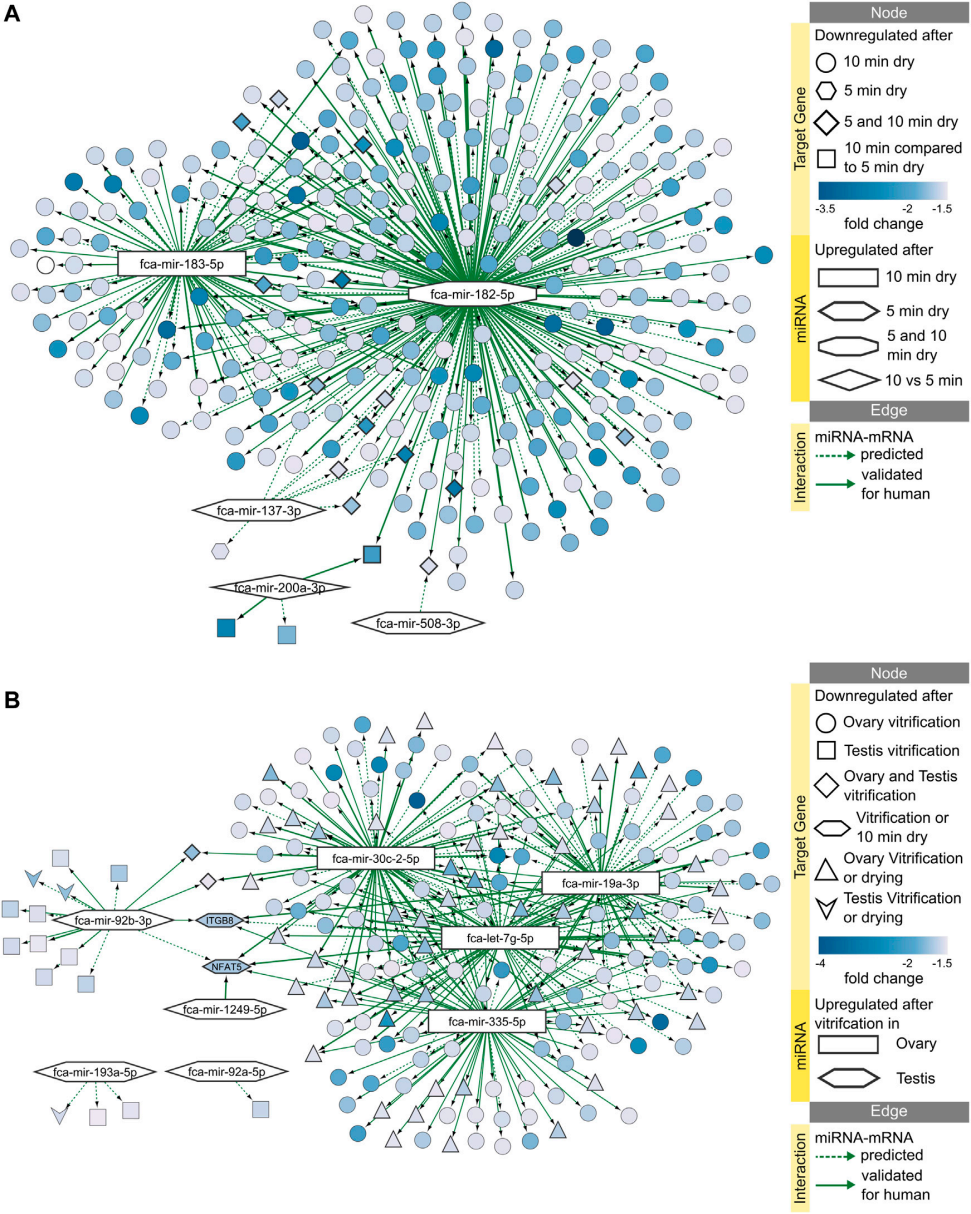
miRNA-mRNA predicted interaction networks responding to preservation protocols. Networks of upregulated miRNAs and downregulated target mRNAs after **(A)** 5 or 10 min dehydration, and **(B)** vitrification of ovarian or testicular tissues. Interactive networks with corresponding data tables are available in Supplementary Data Sheet S3.

We identified several miRNAs that are upregulated in the ovarian tissue in response to either dehydration or vitrification preservation protocols, and in the testicular tissue in response to vitrification (Supplementary Table S3.1). Only testicular tissues from adult showed response of miRNAs to vitrification, while no differentially expressed miRNAs were found in juvenile testis. Using our previous data on RNA-seq in ovarian and testicular tissues undergoing the same preservation protocols (Amelkina and Comizzoli, 2020), we could build in-silico miRNA-mRNA interaction networks for effects of dehydration (Figure 6A; Supplementary Tables S3.2–5) and vitrification (Figure 6B; Supplementary Tables S3.6–7). Both networks can be viewed interactively using files in Supplementary Data Sheet S3. Figure 6A shows that miRNA fca-mir-182-5p plays the biggest role in inhibiting mRNAs in response to both 5- and 10-min dehydration protocols. No upregulated miRNAs are shared between ovarian and testicular tissue response to vitrification, but majority of miRNAs inhibit the same two mRNAs, NFAT5 (nuclear factor of activated T-cells 5) and ITGB8 (integrin subunit beta 8), in both gonads (Figure 6B; Supplementary Tables S3.6–7). Additionally, NFAT5 is also inhibited by upregulated miRNAs in response to 10 min dehydration (Figure 6B).

**FIGURE 7:**
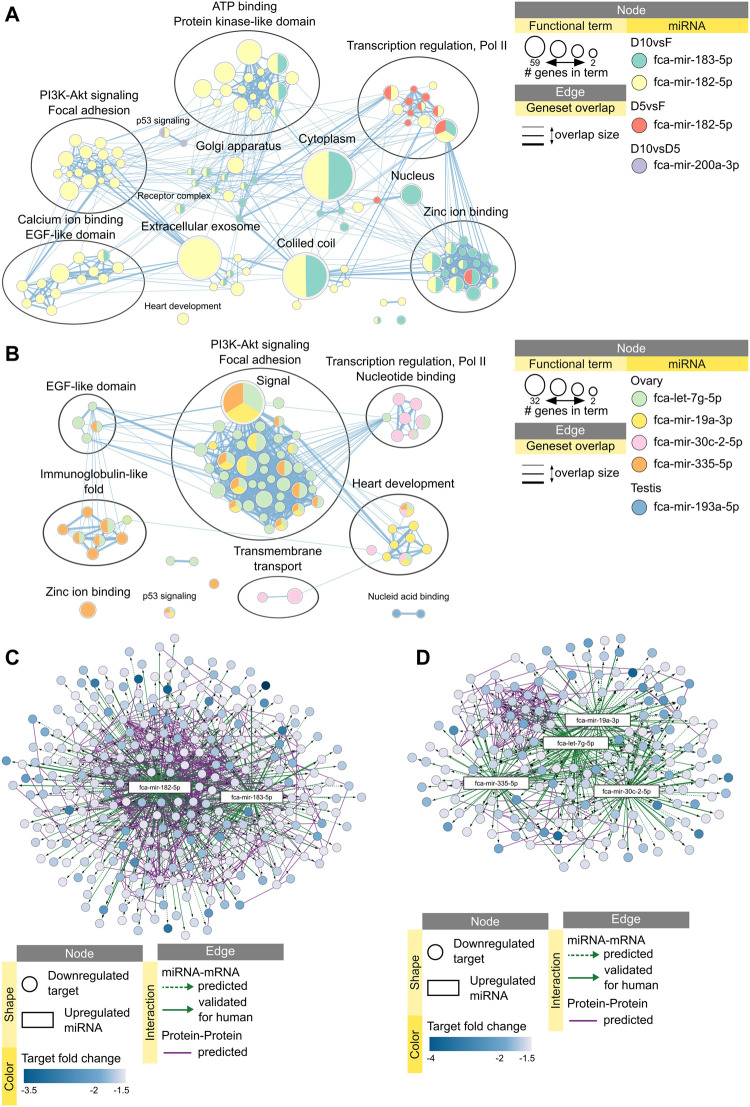
Impact of miRNAs on functional terms, pathway, and protein-protein interactions in response to preservation protocols. Enrichment map displays enriched gene-sets based on downregulated mRNA targets for each upregulated miRNA after **(A)** 5 or 10 min dehydration, and **(B)** vitrification of ovarian or testicular tissues. Interactive networks including networks of each functional cluster with corresponding data tables are available in Supplementary Data Sheet S4. **(C)** miRNA-mRNA-mRNA network for upregulated miRNAs and downregulated mRNAs in response to 10 min dehydration. **(D)** miRNA-mRNA-mRNA network for upregulated miRNAs and downregulated mRNAs in response to vitrification in ovarian tissue. Interactive networks with corresponding data tables are available in Supplementary Data Sheet S3.

Functional enrichment analysis of downregulated mRNA targets predicted that 10 min dehydration inhibit processes of ATP binding, transcription regulation, focal adhesion and PI3K-Akt signaling pathways *via* miRNAs fca-mir-183-5p and fca-mir-182-5p (Figure 7A). Processes of transcription regulation and zinc ion binding are already inhibited after 5 min dehydration *via* fca-mir-182-5p (Figure 7B). Vitrification of ovarian tissues was also predicted to inhibit processes of focal adhesion and PI3K-Akt signaling pathway, but *via* different miRNAs than dehydration protocols (Figure 7B). Vitrification of testicular tissues was predicted to inhibit only nucleic acid binding *via* one miRNA (Figure 7B). Table 3 presents a summary of all miRNAs upregulated in response to dehydration or vitrification, their impact on downregulated mRNAs and inhibited functional clusters. Supplementary Data Sheet S4 provides files for interactive view of networks from Figure 7.

**TABLE 3:** Impact of upregulated miRNAs on downregulated mRNAs after drying or vitrification of gonadal tissue.

miRNA	miRNA family	Gonadal tissue	Upregulated after	Base mean	Fold change	*p*-value	DE targets	Targets, %	DEGs, %	Functional cluster of DE targets
fca-mir-182-5p	mir-182	Ovary	10 min dry	7	2.2	0.0339	256	88.6	42.6	PS, ATP-B, TP, CIB, ZIB
			5 min dry	7	2.5	0.0135	16	94.1	57.1	TP, ZIB
fca-mir-183-5p	mir-183		10 min dry	7	2.5	0.0113	90	31.1	15.0	PS, TP, ZIB
fca-mir-137-3p	mir-137		5 min dry	50	1.5	0.0245	8	47.1	28.6	-
fca-mir-200a-3p	mir-8		10 vs. 5 min dry	7	2.9	0.0090	3	NA	18.7	Term: p53 signaling
fca-let-7g-5p	let-7		Vitrification	69,658	1.4	0.0008	86	48.6	30.7	PS, TP
fca-mir-19a-3p	mir-19			378	1.5	9.47E-05	69	39.0	24.6	PS
fca-mir-30c-2-5p	mir-30			560	1.4	0.0040	81	45.8	28.9	TP
fca-mir-335-5p	mir-335			1,584	1.6	0.0035	70	39.5	25.0	PS
fca-mir-92b-3p	mir-25	Testis		60	1.8	0.0013	15	78.9	31.3	-
fca-mir-92a-5p				55	1.5	0.0297	1	5.3	2.1	-
fca-mir-193a-5p	mir-193			146	1.5	0.0105	3	15.8	6.3	Term: nucleic acid binding
fca-mir-1249-5p	mir-1249			15	1.6	0.0495	1	5.3	2.1	-

Targets, %—percentage of genes regulated by the miRNA out of all targets in the dataset. DEGs, % - percentage of genes impacted by miRNA out of all downregulated genes. DE, differentially expressed (downregulated). Functional clusters: ATP-B, ATP binding; CIB, Calcium ion binding; PS, PI3K-Akt signaling; TP, Transcription, Polymerase II; ZIB, Zinc ion binding.

Finally, we added predicted protein-protein interactions into our miRNA-mRNA networks derived from STRING database of domestic cat to elucidate miRNA-mRNA-mRNA interactions during cellular stress response. We then performed functional enrichment on each predicted protein-protein interaction cluster to further identify functions potentially inhibited *via* miRNAs in response to preservation protocols. Targets downregulated by fca-mir-182-5p and fca-mir-183-5p after 10 min dehydration formed a large, predicted protein-protein interaction cluster (Figure 7C), which was enriched in processes related to focal adhesion and PI3K-Akt signaling pathway (Supplementary Table S3.8). Targets downregulated by miRNAs after vitrification formed a smaller predicted protein-protein interaction cluster, mainly interacting with fca-mir-19a-3p, fca-let-7g-5p and fca-mir-335-5p (Figure 7D) and enriched in focal adhesion and PI3K-Akt signaling pathway (Supplementary Table S3.9).

Overall, 10 min dehydration protocol had the biggest predicted impact of upregulated miRNAs on downregulated mRNAs in the ovarian tissue, with fca-mir-182-5p being upregulated already after 5 min drying, having most predicted interactions with mRNA-mRNA target cluster, and potentially inhibiting focal adhesion and PI3K-Akt signaling pathway (Table 3). In testicular tissues, miRNAs don’t seem to have a high impact on mRNA vitrification response (Table 3).

## Discussion

We identified 235 miRNA precursors in the domestic cat ovarian and testicular tissues, including 183 previously reported for the cat (out of 271) (Laganà et al., 2017) and 52 novel feline candidate miRNA precursors. Identified miRNAs showed tissue-, stage- and condition- specific expression. Clusters of miRNAs united by shared differentially expressed (DE) mRNA targets and function are predicted to be involved in testicular development and spermatogenesis. miRNAs may play a significant role in ovarian tissue response to microwave-assisted dehydration, with smaller roles in cellular response to vitrification in both ovary and testis.

Over the past decade, microRNAs have been shown to be involved in regulation of many biological processes, and specifically participate in multiple levels of gene expression regulation in spermatogenesis and follicular development, such as cell proliferation, cell differentiation, apoptosis and meiosis (García-López et al., 2015; Li et al., 2015; He et al., 2021). In our study, while 95 feline miRNA precursors were uniformly expressed in both testicular and ovarian tissues, the rest showed tissue-specific expression with 67 precursors enriched in the ovary and 83 in testis. In the mouse model, the deficiency of the miRNA processing enzyme Dicer1 led to luteal insufficiency and infertility in female mice (Otsuka et al., 2008), with further studies showing that the observed impaired luteal angiogenesis was partly caused by the deletion of miR-17-5p and let-7b. Both miR-17 and let-7 were expressed in the domestic cat ovary and testis, with let-7e and let-7i being enriched in the ovarian tissue. Previous studies also showed that FSH can regulate the expression of miR-29a and miR-30d in cultured granulosa cells in a time-specific manner (Yao et al., 2010), while the appearance of LH peak can promote the expression of miR-21 and miR-132 in granulosa cells (Fiedler et al., 2008). In our study, the domestic cat ovarian tissue showed to express all these miRNAs at a detectable level, suggesting their potential role in the ovarian function in felids as well. Similarly, multiple miRNAs reported to have function in testis, such as miR-17–92 cluster, miR-20, miR-21, miR-106, miR-24, miR-34 and miR-204 (Bouhallier et al., 2010; Niu et al., 2011, 2016; He et al., 2013; Kotaja, 2014), were expressed in domestic cat testicular tissue. Out of these, miR-106a, miR-204 and miR-34, as well as miRNAs from miR-17–92 cluster, were enriched in testis in comparison to the ovary. MiR-106a and miR-204 have been shown to stimulate the proliferation of mice spermatogonia stem cells (SSCs) (He et al., 2013), while miR-34c can affect apoptosis of male germ cells in goat and mouse (Liang et al., 2012; Li et al., 2013).

Testicular development and spermatogenesis consist of tightly regulated physiological progress that results in production of spermatozoa from SSCs. The process of spermatogenesis includes the production of spermatocytes *via* stem-cell mitosis, which then undergo two meiosis and produce haploid round sperm cells that eventually become mature sperm (Griswold, 2016). The role of miRNAs in spermatogenesis has not been fully elucidated; however, high-throughput expression studies have shown that a large number of miRNAs are selectively expressed in sperm cells at different stages of development, including spermatogonia, pachytene spermatocytes, spermatozoa and mature spermatozoa (Yan et al., 2007; Moritoki et al., 2014).

Additionally, conditional knockout of miRNA processing enzymes Drosha and Dicer in spermatogenic cells of mice testis after birth led to infertility due to impaired spermatogenesis (Wilhelmm and Bernard, 2016). All these findings suggest that miRNAs play a crucial role in regulating spermatogenesis and testis development. Previously, we characterized transcriptome dynamics during testicular development in domestic cats and showed that mRNA expression changes significantly from early to late juvenile period into adulthood, which was also associated with the presence of specific germ cells (Amelkina et al., 2021). In the current study, we used the same samples, except for two adult cats, to be able to map changes in miRNA to the characterized histological and transcriptomic phenotype of each testis. Similarly to our mRNA results (Amelkina et al., 2021), we observed clear separation of adult and juvenile samples based on overall miRNA expression, with the sample from the earliest development stage being separated from both adult and juvenile testes. Differential expression analysis highlighted significant changes in miRNA testicular expression between early and late juvenile and adult cats, suggesting that miRNAs may play an important role in feline testis development and spermatogenesis.

When combining our miRNA data with previously reported mRNA data into miRNA-mRNA interaction networks, we could identify whole clusters of differentially expressed miRNAs that share DE target mRNAs and are further united into functional clusters based on enrichment analysis. These results fit in the prevailing model that posits that miRNAs function by fine-tuning the expression of numerous targets, and mRNA targets are in turn regulated by numerous miRNAs. While each target is regulated subtly, the additive effect of coordinated regulation of a large suite of transcripts is believed to result in strong phenotypic outputs. Additionally, miRNAs show redundancy in function which may arise from the acquired overlapping sets of targets (Mendell and Olson, 2012).

One of the biggest miRNA clusters upregulated from juvenile to adult testis included 16 mature miRNAs: miR-146a-5p, -146b-5p, -15b-3p/5p, -29b-2-3p, -29c-3p, -449-3p/5p, -34c-3p/5p, -34b-3p, -191-3p/5p, -92b-3p/5p, and -200c-3p. This cluster was predicted to inhibit functions in cell-matrix adhesion, morphogenesis, Wnt signaling, immune response, stem cell pluripotency and cell migration in adult compared to early juvenile testis, all of which were reported to also be inhibited in our previous study (Amelkina et al., 2021). From this cluster, miR-34c-3p and miR-200c-3p exhibited continuous upregulation from early to late juvenile to adult period. MicroRNA-34 is highly conserved in evolution and its miR-34 family has been identified to be pivotal in organ development, organismal or organ senescence, stress response, spermatogenesis and signal transduction (Wang et al., 2022). In goat testis, miR-34c has been shown to promote apoptosis and suppress proliferation of SSCs (Li et al., 2013). Silencing of miR-34c in mice primary spermatocytes prevented germ cells from apoptosis induced by deprivation of testosterone (Liang et al., 2012). Microarray study in human pachytene spermatocytes, round spermatids and Sertoli cells showed that miR-34c was highly expressed specifically in germ cells, and could play a role by enhancing the germinal phenotype of cells already committed to this lineage (Bouhallier et al., 2010). Continuous increase in expression of miR-34c from early to late juvenile to adult period, which also coincided with the increase in the presence of spermatocytes and spermatids, suggests that miR-34c may play an important role in domestic cat spermatogenesis as well. Interestingly, NOTCH2 has been shown to be a direct target of miR-34c with its expression greatly decreased in miR34c transfected cells (Bouhallier et al., 2010). In our study, NOTCH2 paralog NOTCH1 has been predicted as a DE target of several miRNAs from the major cluster, including miR-34b-3p, miR-34c-5p, miR-146a-5p, miR-200c-3p, miR-449-5p, and miR-92b-3p.

Tissue preservation protocols, such as vitrification, slow-freezing and recently developed microwave-assisted dehydration, expose cells to non-physiological conditions which may disturb cellular homeostasis and lead to cellular stress (Lee et al., 2019; Amelkina and Comizzoli, 2020). Protocols are constantly being optimized to reduce the severity of cellular stress and mitigate any potential damage to proteins, DNA, RNA, and lipids. In response to stress, cells attempt to restore cellular homeostasis or adapt to environmental conditions using such mechanisms as growth arrest, repair or clearance of damaged macromolecules, as well as changes in the gene expression patterns (Olejniczak et al., 2018).

Growing amount of studies have been supporting the role of miRNAs in the response of tissues to physiologic and pathophysiologic stress (Leung and Sharp, 2010; Mendell and Olson, 2012; Zhang and Peng, 2015). microRNAs can target multiple transcripts at the same time and exert their functions without the need to synthesize proteins, therefore providing fast and economical way to regulate stress response. By targeting multiple transcription factors and other signaling molecules, miRNAs can induce significant physiological effect during stress even at subtle expression levels (Gosline et al., 2016).

In our study, the biggest effect on miRNA expression was caused by drying and rehydrating protocol of microwave-assisted dehydration technique, leading to upregulation of miR-183-5p and miR-182-5p, both of which formed a cluster *via* shared DE mRNA targets and enriched functions. Interestingly, miR-182-5p and miR-183-5p are known to be part of a miR-182/183/96 cluster with a distance of 4 kb from each other on the mouse chromosome 6qA3, being transcribed together (Zhang et al., 2015). In the domestic cat, miR-182 and miR-183 are also located on the same chromosome chrA2 with 4 kb distance from each other, suggesting they might be also transcribed together as a cluster in felids. Both miR-182-5p and miR-183-5p have been shown to be involved regulating proliferation of cancer cells *via* inhibition of apoptosis and autophagy (Zhang et al., 2015, 2021; Huangfu et al., 2016). Additionally, miR-182-5p has been shown to inhibit oxidative stress in RAW264.7 cells (Qin et al., 2018) and protect human lens epithelial cells against oxidative stress-induced apoptosis by inhibiting p38 MAPK signaling (Li et al., 2020). In our previous study, we have shown that ovarian tissues did not exhibit signs of apoptosis after dehydration and rehydration (Lee et al., 2019; Amelkina and Comizzoli, 2020). Moreover, in the current study miR-182-5p was predicted to downregulate mRNA targets enriched in signaling pathways, such as PI3K-Akt. Our results suggest that miR-182-5p and miR-183-5p are transcribed in cluster in ovarian tissues in response to dehydration and/or rehydration induced stress and potentially protect the cells from oxidative-stress injury and apoptosis activation.

microRNAs exert pleiotropic nature, have ATP-inexpensive production, rapid action and reversible regulation of mRNA function, all of which makes them an effective drug target (Hadj-Moussa et al., 2022). A major insight into miRNA functions under stress conditions have been obtained from studies on survival strategies of stress-tolerant animals. All stress-tolerant animals undergo metabolic reorganization as part of their adaptive strategies, including hibernation, freeze tolerance, hypoxia and anoxia tolerance (Storey and Storey, 2017; Hadj-Moussa et al., 2022).

In our study, we identified changes in expression of several miRNAs in response to vitrification stress that have also been reported to be involved in the strategies of stress-tolerant animals. Like this, miR-335 has been shown to regulate hypoxia-inducible transcription factor 1 in brains of hypoxic naked mole rats (Li et al., 2015; Hadj-Moussa et al., 2021), while in our study it was upregulated in ovarian tissues after vitrification and warming. A study on hibernating grey mouse lemurs reported increased expression of miRNAs miR-92a and miR-193b during torpor, which was related to regulation of p53 signaling and mTOR pathway (Li et al., 2017; Biggar et al., 2018; Zhang et al., 2020). In our study, these miRNAs were upregulated in testicular tissues after vitrification and warming. Finally, a subgroup of miRNAs termed mitochondrial miRNAs have been linked with various mitochondrial functions ranging from energy metabolism to apoptosis (Borralho et al., 2015). Two miRNAs in our study, let-7g-5p and miR-19a-3p, that share a family with some of the reported mitochondrial miRNAs, let-7b and miR-19b, were upregulated in the ovarian tissue in response to vitrification and warming. Interestingly, our previous study also showed predicted adaptive changes in mitochondrial respiration in response to vitrification in the ovary (Amelkina and Comizzoli, 2020). We may hypothesize, that some of these adaptive gene expression changes are regulated by miRNAs, in particular the identified cluster of miR-30c-2-5p, miR-19a-3p, miR-335-5p and let-7g-5p.

In our previous study we hypothesized that testicular tissue response to freezing and warming stress might be similar to ischemia (Amelkina et al., 2021). In our current study, we observed the upregulation of miR-92a-5p and miR-92b-3p in vitrified testicular tissues compared to fresh. Interestingly, miR-92a has been shown to be upregulated by ischemia as a negative regulator of blood vessel growth. Systemic delivery of antisense oligonucleotides directed against miR-92a in a mouse model of hindlimb ischemia increased blood vessel growth and improved recovery from ischemic damage (Bonauer et al., 2009). Potentially, miR-92 may also be involved in ischemic response in cat testis and manipulation of its expression might be one of the future considerations in improvement of testicular vitrification protocols.

One of the limitations of our study was exclusion of additional ovarian and testicular groups from the experiment. We included both juvenile and adult testis, but only prepubertal ovary into the study, because the pool of primary and primordial follicles which we collected with the ovarian cortex remains into cat puberty. In contrast, the testicular tissue undergoes dramatic changes during development and puberty, resulting in shift of cellular content and emergence of new cells. We also did not add the group of dehydrated testicular tissues, because the microwave-assisted dehydration protocol is only in its first development step for the testis (Silva et al., 2020) and has not been optimized like the ovarian one (Lee et al., 2019). In future studies, investigating miRNA-mRNA expression at different stages of follicular cycle would add an important puzzle piece to the whole picture of miRNAs in domestic cat gonads. Additionally, we only measured transcriptomic response within 30 min of tissue reanimation, but it is possible that we can see more changes in miRNA and gene expression after *in vitro* culturing. For example, mature miRNAs can accumulate later than immediately transcribed pri-miRNAs; this delay acts as the timer for stress response during inflammation (Olejniczak et al., 2018). As our next steps, it is important to optimize the ovarian and testicular culture conditions to measure the effect at least 24 h after tissue reanimation. Our datasets for miRNA and mRNA come mostly from the same samples, however, it is still important to validate the *in-silico* miRNA-mRNA networks we predicted *via* targeted gene expression studies. Finally, there are many levels at which mRNA expression can be modulated and miRNAs are only one part of the puzzle. Future studies should focus on additional factors of gene expression regulation, as well as more functional studies of identified miRNAs with localization of genes and proteins of interest in ovarian and testicular tissues.

In conclusion, we performed an extensive discovery of miRNAs in the ovarian and testicular tissues of the domestic cat, which resulted in additional 52 novel feline candidate miRNA precursors and identification of ovary- and testis-enriched miRNAs. Integrating the mRNA data from our previous reports, we could create the in-silico miRNA-mRNA interaction networks which revealed that miRNAs might function in clusters of similar targets and functions in testis development. The list of miRNAs involved in the response of gonadal tissues to vitrification and microwave-assisted dehydration adds to our understanding of the involvement of miRNAs in various cellular stress responses, as well as prediction of miRNA targets for protocol improvements. Obtained list of miRNAs that are enriched in the ovary or testis, participate in testicular development, and have a potential role in cellular stress response is an important step towards deciphering complex mechanisms and networks involved in biological processes in feline gonadal tissues with the ultimate goal of understanding the gonadal biology. Our results also contribute to optimization of preservation protocols and identification of miRNA biomarkers for the development of assisted reproduction techniques in felids.

## Data Availability

The datasets presented in this study can be found in online repositories. The names of the repository/repositories and accession number(s) can be found in the article/Supplementary Material.

## References

[B1] Amelkina O. Comizzoli P. (2020). Initial response of ovarian tissue transcriptome to vitrification or microwave-assisted dehydration in the domestic cat model. BMC Genomics 21, 828. 10.1186/s12864-020-07236-z 33238878 PMC7690003

[B2] Amelkina O. Silva A. M. Comizzoli P. (2021). Transcriptome dynamics in developing testes of domestic cats and impact of age on tissue resilience to cryopreservation. BMC Genomics 22, 847. 10.1186/s12864-021-08099-8 34814833 PMC8611880

[B3] An J. Lai J. Lehman M. L. Nelson C. C. (2013). miRDeep*: an integrated application tool for miRNA identification from RNA sequencing data. Nucleic Acids Res. 41, 727–737. 10.1093/nar/gks1187 23221645 PMC3553977

[B4] Barberán-Soler S. Vo J. M. Hogans R. E. Dallas A. Johnston B. H. Kazakov S. A. (2018). Decreasing miRNA sequencing bias using a single adapter and circularization approach. Genome Biol. 19, 105. 10.1186/s13059-018-1488-z 30173660 PMC6120088

[B5] Biggar K. K. Luu B. E. Wu C. W. Pifferi F. Perret M. Storey K. B. (2018). Identification of novel and conserved microRNA and their expression in the gray mouse lemur, *Microcebus murinus*, a primate capable of daily torpor. Gene 677, 332–339. 10.1016/j.gene.2018.08.014 30103007

[B6] Bonauer A. Carmona G. Iwasaki M. Mione M. Koyanagi M. Fischer A. (2009). MicroRNA-92a controls angiogenesis and functional recovery of ischemic tissues in mice. Science 324, 1710–1713. 10.1126/science.1174381 19460962

[B7] Borralho P. M. Rodrigues C. M. P. Steer C. J. (2015). microRNAs in mitochondria: An unexplored niche. microRNA Basic Science,Advances Exp. Med. Biol., 31–51. 10.1007/978-3-319-22380-3_3 26662985

[B8] Bouhallier F. Allioli N. Lavial F. Chalmel F. Perrard M.-H. Durand P. (2010). Role of miR-34c microRNA in the late steps of spermatogenesis. RNA 16, 720–731. 10.1261/rna.1963810 20150330 PMC2844620

[B9] Catalanotto C. Cogoni C. Zardo G. (2016). MicroRNA in control of gene expression: An overview of nuclear functions. Ijms 17, 1712. 10.3390/ijms17101712 PMC5085744 27754357

[B10] Cline M. S. Smoot M. Cerami E. Kuchinsky A. Landys N. Workman C. (2007). Integration of biological networks and gene expression data using Cytoscape. Nat. Protoc. 2, 2366–2382. 10.1038/nprot.2007.324 17947979 PMC3685583

[B11] Comizzoli P. (2015). Biobanking efforts and new advances in male fertility preservation for rare and endangered species. Asian J. Androl. 17, 640–645. 10.4103/1008-682X.153849 25966625 PMC4492057

[B12] Comizzoli P. Songsasen N. Wildt D. E. (2010). Protecting and extending fertility for females of wild and endangered mammals. Cancer Treat. Res. 156, 87–100. 10.1007/978-1-4419-6518-9_7 20811827 PMC3086462

[B13] Cong W. Zhang X.-X. He J.-J. Li F.-C. Elsheikha H. M. Zhu X.-Q. (2017). Global miRNA expression profiling of domestic cat livers following acute Toxoplasma gondii infection. Oncotarget 8, 25599–25611. 10.18632/oncotarget.16108 28424428 PMC5421954

[B14] da Silveira W. A. Renaud L. Simpson J. Glen W. B. Hazard E. S. Chung D. (2018). miRmapper: A tool for interpretation of miRNA-mRNA interaction networks. Genes 9, 458. 10.3390/genes9090458 PMC6162471 30223528

[B15] Dai D.-H. Qazi I. H. Ran M.-X. Liang K. Zhang Y. Zhang M. (2019). Exploration of miRNA and mRNA profiles in fresh and frozen-thawed boar sperm by transcriptome and small RNA sequencing. Ijms 20, 802. 10.3390/ijms20040802 PMC6413023 30781801

[B16] Daneshvar M. Movahedin M. Salehi M. Noruzinia M. (2021). Alterations of miR-16, miR-let-7a and their target genes expression in human blastocysts following vitrification and re-vitrification. Reprod. Biol. Endocrinol. 19, 155. 10.1186/s12958-021-00842-w 34627262 PMC8501585

[B17] Doncheva N. T. Morris J. H. Gorodkin J. Jensen L. J. (2019). Cytoscape StringApp: Network analysis and visualization of proteomics data. J. Proteome Res. 18, 623–632. 10.1021/acs.jproteome.8b00702 30450911 PMC6800166

[B18] Fiedler S. D. Carletti M. Z. Hong X. Christenson L. K. (2008). Hormonal regulation of MicroRNA expression in periovulatory mouse mural granulosa Cells1. Biol. Reprod. 79, 1030–1037. 10.1095/biolreprod.108.069690 18716288 PMC2780477

[B19] Fleischhacker S. N. Bauersachs S. Wehner A. Hartmann K. Weber K. (2013). Differential expression of circulating microRNAs in diabetic and healthy lean cats. Veterinary J. 197, 688–693. 10.1016/j.tvjl.2013.03.027 23636037

[B20] Friedländer M. R. Chen W. Adamidi C. Maaskola J. Einspanier R. Knespel S. (2008). Discovering microRNAs from deep sequencing data using miRDeep. Nat. Biotechnol. 26, 407–415. 10.1038/nbt1394 18392026

[B21] Friedman R. C. Farh K. K.-H. Burge C. B. Bartel D. P. (2009). Most mammalian mRNAs are conserved targets of microRNAs. Genome Res. 19, 92–105. 10.1101/gr.082701.108 18955434 PMC2612969

[B22] García-López J. Alonso L. Cárdenas D. B. Artaza-Alvarez H. Hourcade J. de D. Martínez S. (2015). Diversity and functional convergence of small noncoding RNAs in male germ cell differentiation and fertilization. RNA 21, 946–962. 10.1261/rna.048215.114 25805854 PMC4408801

[B23] Gosline S. J. C. Gurtan A. M. JnBaptiste C. K. Bosson A. Milani P. Dalin S. (2016). Elucidating MicroRNA regulatory networks using transcriptional, post-transcriptional, and histone modification measurements. Cell Rep. 14, 310–319. 10.1016/j.celrep.2015.12.031 26748710 PMC4831719

[B24] Griffiths-Jones S. Moxon S. Marshall M. Khanna A. Eddy S. R. Bateman A. (2004). Rfam: Annotating non-coding RNAs in complete genomes. Nucleic Acids Res. 33, D121–D124. 10.1093/nar/gki081 PMC540035 15608160

[B25] Griswold M. D. (2016). Spermatogenesis: The commitment to meiosis. Physiol. Rev. 96, 1–17. 10.1152/physrev.00013.2015 26537427 PMC4698398

[B26] Gruber A. R. Lorenz R. Bernhart S. H. Neubock R. Hofacker I. L. (2008). The Vienna RNA websuite. Nucleic Acids Res. 36, W70–W74. 10.1093/nar/gkn188 18424795 PMC2447809

[B27] Ha M. Kim V. N. (2014). Regulation of microRNA biogenesis. Nat. Rev. Mol. Cell Biol. 15, 509–524. 10.1038/nrm3838 25027649

[B28] Hadj-Moussa H. Hawkins L. J. Storey K. B. (2022). Role of MicroRNAs in extreme animal survival strategies. Methods Mol. Biology,miRNomics, 311–347. 10.1007/978-1-0716-1170-8_16 34432286

[B29] Hadj-Moussa H. Pamenter M. E. Storey K. B. (2021). Hypoxic naked mole-rat brains use microRNA to coordinate hypometabolic fuels and neuroprotective defenses. J. Cell Physiol. 236, 5080–5097. 10.1002/jcp.30216 33305831

[B30] Hamada M. Kiryu H. Sato K. Mituyama T. Asai K. (2009). Prediction of RNA secondary structure using generalized centroid estimators. Bioinformatics 25, 465–473. 10.1093/bioinformatics/btn601 19095700

[B31] He C. Wang K. Gao Y. Wang C. Li L. Liao Y. (2021). Roles of noncoding RNA in reproduction. Front. Genet. 12, 777510. 10.3389/fgene.2021.777510 34956326 PMC8695933

[B32] He Z. Jiang J. Kokkinaki M. Tang L. Zeng W. Gallicano I. (2013). MiRNA-20 and mirna-106a regulate spermatogonial stem cell renewal at the post-transcriptional level via targeting STAT3 and Ccnd1. Stem Cells 31, 2205–2217. 10.1002/stem.1474 23836497 PMC3859454

[B33] Heidari F. Hosseini S. Yeganeh S. M. Salehi M. (2019). Expression of miR-Let-7a, miR-15a, miR-16-1, and their target genes in fresh and vitrified embryos and its surrounding culture media for noninvasive embryo assessment. J Cell. Biochem. 120, 19691–19698. 10.1002/jcb.29275 31297859

[B34] Huang D. W. Sherman B. T. Lempicki R. A. (2009). Systematic and integrative analysis of large gene lists using DAVID bioinformatics resources. Nat. Protoc. 4, 44–57. 10.1038/nprot.2008.211 19131956

[B35] Huangfu L. Liang H. Wang G. Su X. Li L. Du Z. (2016). miR-183 regulates autophagy and apoptosis in colorectal cancer through targeting of UVRAG. Oncotarget 7, 4735–4745. 10.18632/oncotarget.6732 26717041 PMC4826239

[B36] Kim V. N. Han J. Siomi M. C. (2009). Biogenesis of small RNAs in animals. Nat. Rev. Mol. Cell Biol. 10, 126–139. 10.1038/nrm2632 19165215

[B37] Kotaja N. (2014). MicroRNAs and spermatogenesis. Fertil. Steril. 101, 1552–1562. 10.1016/j.fertnstert.2014.04.025 24882619

[B38] Kozomara A. Griffiths-Jones S. (2014). miRBase: annotating high confidence microRNAs using deep sequencing data. Nucl. Acids Res. 42, D68–D73. 10.1093/nar/gkt1181 24275495 PMC3965103

[B39] Krol J. Loedige I. Filipowicz W. (2010). The widespread regulation of microRNA biogenesis, function and decay. Nat. Rev. Genet. 11, 597–610. 10.1038/nrg2843 20661255

[B40] Laganà A. Dirksen W. P. Supsavhad W. Yilmaz A. S. Ozer H. G. Feller J. D. (2017). Discovery and characterization of the feline miRNAome. Sci. Rep. 7, 9263. 10.1038/s41598-017-10164-w 28835705 PMC5569061

[B41] Langmead B. Trapnell C. Pop M. Salzberg S. L. (2009). Ultrafast and memory-efficient alignment of short DNA sequences to the human genome. Genome Biol. 10, R25. 10.1186/gb-2009-10-3-r25 19261174 PMC2690996

[B42] Lee P.-C. Adams D. M. Amelkina O. White K. K. Amoretti L. A. Whitaker M. G. (2019). Influence of microwave-assisted dehydration on morphological integrity and viability of cat ovarian tissues: First steps toward long-term preservation of complex biomaterials at supra-zero temperatures. PLoS One 14, e0225440. 10.1371/journal.pone.0225440 31800613 PMC6892495

[B43] Leung A. K. L. Sharp P. A. (2010). MicroRNA functions in stress responses. Mol. Cell 40, 205–215. 10.1016/j.molcel.2010.09.027 20965416 PMC2996264

[B44] Lewis B. P. Burge C. B. Bartel D. P. (2005). Conserved seed pairing, often flanked by adenosines, indicates that thousands of human genes are microRNA targets. Cell 120, 15–20. 10.1016/j.cell.2004.12.035 15652477

[B45] Li C. Chen Y. Chen X. Wei Q. Cao B. Shang H. (2017). Downregulation of MicroRNA-193b-3p promotes autophagy and cell survival by targeting TSC1/mTOR signaling in NSC-34 cells. Front. Mol. Neurosci. 10. 10.3389/fnmol.2017.00160 PMC5447700 28611587

[B46] Li J. Yang X. Liu F. Song Y. Liu Y. (2019). Evaluation of differentially expressed microRNAs in vitrified oocytes by next generation sequencing. Int. J. Biochem. Cell Biol. 112, 134–140. 10.1016/j.biocel.2019.05.006 31108211

[B47] Li M. Yu M. Liu C. Zhu H. He X. Peng S. (2013). miR-34c works downstream of p53 leading to dairy goat male germline stem-cell ( mGSC s) apoptosis. Cell Prolif. 46, 223–231. 10.1111/cpr.12013 23510477 PMC6495960

[B48] Li Y. Fang Y. Liu Y. Yang X. (2015). MicroRNAs in ovarian function and disorders. J. Ovarian Res. 8, 51. 10.1186/s13048-015-0162-2 26232057 PMC4522283

[B49] Li Z.-N. Ge M.-X. Yuan Z.-F. (2020). MicroRNA-182-5p protects human lens epithelial cells against oxidative stress-induced apoptosis by inhibiting NOX4 and p38 MAPK signalling. BMC Ophthalmol. 20, 233. 10.1186/s12886-020-01489-8 32552665 PMC7301500

[B50] Liang X. Zhou D. Wei C. Luo H. Liu J. Fu R. (2012). MicroRNA-34c enhances murine male germ cell apoptosis through targeting ATF1. PLoS One 7, e33861. 10.1371/journal.pone.0033861 22479460 PMC3316505

[B51] Lima D. B. C. Silva L. D. M. Comizzoli P. (2018). Influence of warming and reanimation conditions on seminiferous tubule morphology, mitochondrial activity, and cell composition of vitrified testicular tissues in the domestic cat model. PLOS ONE 13, e0207317. 10.1371/journal.pone.0207317 30408126 PMC6224116

[B52] Love M. I. Huber W. Anders S. (2014). Moderated estimation of fold change and dispersion for RNA-seq data with DESeq2. Genome Biol. 15, 550. 10.1186/s13059-014-0550-8 25516281 PMC4302049

[B53] Lyons P. J. Lang-Ouellette D. Morin P. (2013). CryomiRs: Towards the identification of a cold-associated family of microRNAs. Comp. Biochem. Physiology Part D Genomics Proteomics 8, 358–364. 10.1016/j.cbd.2013.10.001 24212287

[B54] Mackowiak S. D. (2011). Identification of novel and known miRNAs in deep-sequencing data with miRDeep2. Curr. Protoc. Bioinforma. 36, 1. 10.1002/0471250953.bi1210s36 22161567

[B55] Martin M. (2011). Cutadapt removes adapter sequences from high-throughput sequencing reads. EMBnet J. 17, 10–12. 10.14806/ej.17.1.200

[B56] Mendell J. T. Olson E. N. (2012). MicroRNAs in stress signaling and human disease. Cell 148, 1172–1187. 10.1016/j.cell.2012.02.005 22424228 PMC3308137

[B57] Merico D. Isserlin R. Stueker O. Emili A. Bader G. D. (2010). Enrichment map: A network-based method for gene-set enrichment visualization and interpretation. PLoS One 5, e13984. 10.1371/journal.pone.0013984 21085593 PMC2981572

[B58] Moritoki Y. Hayashi Y. Mizuno K. Kamisawa H. Nishio H. Kurokawa S. (2014). Expression profiling of microRNA in cryptorchid testes: miR-135a contributes to the maintenance of spermatogonial stem cells by regulating FoxO1. J. Urology 191, 1174–1180. 10.1016/j.juro.2013.10.137 24184258

[B59] Mouttham L. Comizzoli P. (2016). The preservation of vital functions in cat ovarian tissues during vitrification depends more on the temperature of the cryoprotectant exposure than on the sucrose supplementation. Cryobiology 73, 187–195. 10.1016/j.cryobiol.2016.07.013 27475292

[B60] Movahed E. Soleimani M. Hosseini S. Akbari Sene A. Salehi M. (2019). Aberrant expression of miR-29a/29b and methylation level of mouse embryos after *in vitro* fertilization and vitrification at two-cell stage. J. Cell. Physiology 234, 18942–18950. 10.1002/jcp.28534 30916357

[B61] Niu B. Wu J. Mu H. Li B. Wu C. He X. (2016). miR-204 regulates the proliferation of dairy goat spermatogonial stem cells via targeting to Sirt1. Rejuvenation Res. 19, 120–130. 10.1089/rej.2015.1719 26213858

[B62] Niu Z. Goodyear S. M. Rao S. Wu X. Tobias J. W. Avarbock M. R. (2011). MicroRNA-21 regulates the self-renewal of mouse spermatogonial stem cells. Proc. Natl. Acad. Sci. U.S.A. 108, 12740–12745. 10.1073/pnas.1109987108 21768389 PMC3150879

[B63] O'Brien S. J. Menotti-Raymond M. Murphy W. J. Yuhki N. (2002). The feline genome project. Annu. Rev. Genet. 36, 657–686. 10.1146/annurev.genet.36.060602.145553 12359739

[B64] Olejniczak M. Kotowska-Zimmer A. Krzyzosiak W. (2018). Stress-induced changes in miRNA biogenesis and functioning. Cell. Mol. Life Sci. 75, 177–191. 10.1007/s00018-017-2591-0 28717872 PMC5756259

[B65] Otsuka M. Zheng M. Hayashi M. Lee J.-D. Yoshino O. Lin S. (2008). Impaired microRNA processing causes corpus luteum insufficiency and infertility in mice. J. Clin. Invest. 118, 1944–1954. 10.1172/JCI33680 18398510 PMC2289794

[B66] Pontius J. U. Mullikin J. C. Smith D. R. Lindblad-Toh A. S. Gnerre K. Clamp S. (2007). Initial sequence and comparative analysis of the cat genome. Genome Res. 17, 1675–1689. 10.1101/gr.6380007 17975172 PMC2045150

[B67] Qin S.-B. Peng D.-Y. Lu J.-M. Ke Z.-P. (2018). MiR-182-5p inhibited oxidative stress and apoptosis triggered by oxidized low-density lipoprotein via targeting toll-like receptor 4. J. Cell Physiol. 233, 6630–6637. 10.1002/jcp.26389 29226948

[B68] Ru Y. Kechris K. J. Tabakoff B. Hoffman P. Radcliffe R. A. Bowler R. (2014). The multiMiR R package and database: Integration of microRNA-target interactions along with their disease and drug associations. Nucleic Acids Res. 42, e133. 10.1093/nar/gku631 25063298 PMC4176155

[B69] Shangguan A. Zhou H. Sun W. Ding R. Li X. Liu J. (2020). Cryopreservation induces alterations of miRNA and mRNA fragment profiles of bull sperm. Front. Genet. 11, 419. 10.3389/fgene.2020.00419 32431726 PMC7214931

[B70] Silva H. V. R. da Silva A. M. Lee P.-C. Brito B. F. Silva A. R. da Silva L. D. M. (2020). Influence of microwave-assisted drying on structural integrity and viability of testicular tissues from adult and prepubertal domestic cats. Biopreservation Biobanking 18, 415–424. 10.1089/bio.2020.0048 32780644

[B71] Singh G. Storey K. B. (2021). MicroRNA cues from nature: A roadmap to decipher and combat challenges in human health and disease? Cells 10, 3374. 10.3390/cells10123374 34943882 PMC8699674

[B72] Storey K. B. Storey J. M. (2017). Molecular physiology of freeze tolerance in vertebrates. Physiol. Rev. 97, 623–665. 10.1152/physrev.00016.2016 28179395

[B73] Sun J. Wang J. Wang S. Yuan D. Birame B. M. Li Z. (2014). MicroRNA profile analysis of a feline kidney cell line before and after infection with mink enteritis virus. Gene 539, 224–229. 10.1016/j.gene.2014.01.074 24525403

[B74] Szklarczyk D. Morris J. H. Cook H. Kuhn M. Wyder S. Simonovic M. (2017). The STRING database in 2017: Quality-controlled protein-protein association networks, made broadly accessible. Nucleic Acids Res. 45, D362–D368. 10.1093/nar/gkw937 27924014 PMC5210637

[B75] Tamazian G. Simonov S. Dobrynin P. Makunin A. Logachev A. Komissarov A. (2014). Annotated features of domestic cat - *Felis catus* genome. GigaSci 3, 2047–217X. 10.1186/2047-217X-3-13 PMC4138527 25143822

[B76] Walker W. H. (2022). Regulation of mammalian spermatogenesis by miRNAs. Seminars Cell & Dev. Biol. 121, 24–31. 10.1016/j.semcdb.2021.05.009 PMC8591147 34006455

[B77] Wang C. Jia Q. Guo X. Li K. Chen W. Shen Q. (2022). microRNA-34 family: From mechanism to potential applications. Int. J. Biochem. Cell Biol. 144, 106168. 10.1016/j.biocel.2022.106168 35085803

[B78] Weber K. Rostert N. Bauersachs S. Wess G. (2015). Serum microRNA profiles in cats with hypertrophic cardiomyopathy. Mol. Cell Biochem. 402, 171–180. 10.1007/s11010-014-2324-8 25573325

[B79] Wilhelmm D. Bernard P. (2016). Non-coding RNAs and the reproductive system. Adv. Exp. Med. Biol. 886, v–vi. 27088146

[B80] Xu X. Li W. Zhang L. Ji Y. Qin J. Wang L. (2021). Effect of sperm cryopreservation on miRNA expression and early embryonic development. Front. Cell Dev. Biol. 9, 749486. 10.3389/fcell.2021.749486 35004670 PMC8728010

[B81] Yan N. Lu Y. Sun H. Tao D. Zhang S. Liu W. (2007). A microarray for microRNA profiling in mouse testis tissues. Reproduction 134, 73–79. 10.1530/REP-07-0056 17641090

[B82] Yao N. Yang B.-Q. Liu Y. Tan X.-Y. Lu C.-L. Yuan X.-H. (2010). Follicle-stimulating hormone regulation of microRNA expression on progesterone production in cultured rat granulosa cells. Endocr 38, 158–166. 10.1007/s12020-010-9345-1 20734245

[B83] Zhang C. Peng G. (2015). Non-coding RNAs: An emerging player in DNA damage response. Mutat. Research/Reviews Mutat. Res. 763, 202–211. 10.1016/j.mrrev.2014.11.003 25795121

[B84] Zhang J. Storey K. B. (2018). RBiomirGS: An all-in-one miRNA gene set analysis solution featuring target mRNA mapping and expression profile integration. PeerJ 6, e4262. 10.7717/peerj.4262 29340253 PMC5768164

[B85] Zhang Q. Ren W. Huang B. Yi L. Zhu H. (2015). MicroRNA-183/182/96 cooperatively regulates the proliferation of colon cancer cells. Mol. Med. Rep. 12, 668–674. 10.3892/mmr.2015.3376 25695717

[B86] Zhang S. Li D. Zhao M. Yang F. Sang C. Yan C. (2021). Exosomal miR-183-5p shuttled by M2 polarized tumor-associated macrophage promotes the development of colon cancer via targeting THEM4 mediated PI3K/AKT and NF-κB pathways. Front. Oncol. 11, 1973. 10.3389/fonc.2021.672684 PMC8267908 34249713

[B87] Zhang S. Yu J. Sun B. Hou G. Yu Z.-J. Luo H. (2020). MicroRNA-92a targets SERTAD3 and regulates the growth, invasion, and migration of prostate cancer cells via the P53 pathway. OncoTargets Ther. 13, 5495–5514. 10.2147/OTT.S249168 PMC7298502 32606766

[B88] Zhang Y. Dai D. Chang Y. Li Y. Zhang M. Zhou G. (2017). Cryopreservation of boar sperm induces differential microRNAs expression. Cryobiology 76, 24–33. 10.1016/j.cryobiol.2017.04.013 28478125

