# Supplementary material for: Feline microRNAome in ovary and testis: Exploration of in-silico miRNA-mRNA networks involved in gonadal function and cellular stress response
Source: Front Genet. 2022 Sep 26;13:1009220. doi: 10.3389/fgene.2022.1009220 (PMC9548565; doi:10.3389/fgene.2022.1009220)
Supplement: Supplementary file 4 [file Presentation1.zip › Supplementary Figures.pdf]

Supplemetary Figure S1

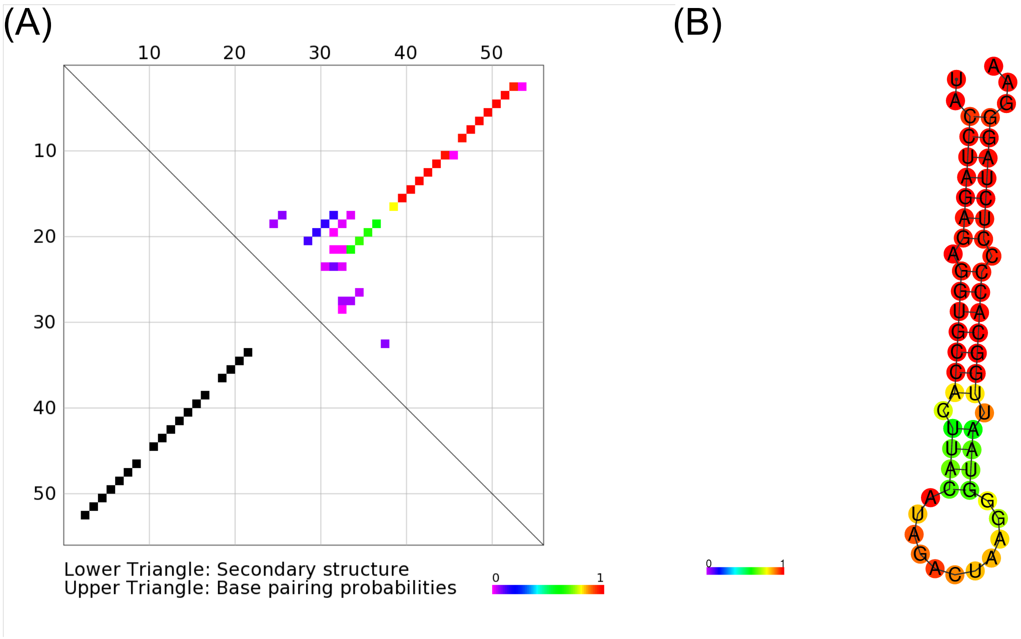

Supplementary Figure S1. Hairpin formation of novel miRNA fca-mir-chrX_QO3CD.
(A) Probability of base pairing. (B) Predicted secondary structure.
Generated using CentroidFold tool at http://rtools.cbrc.jp/.

Supplemetary Figure S2

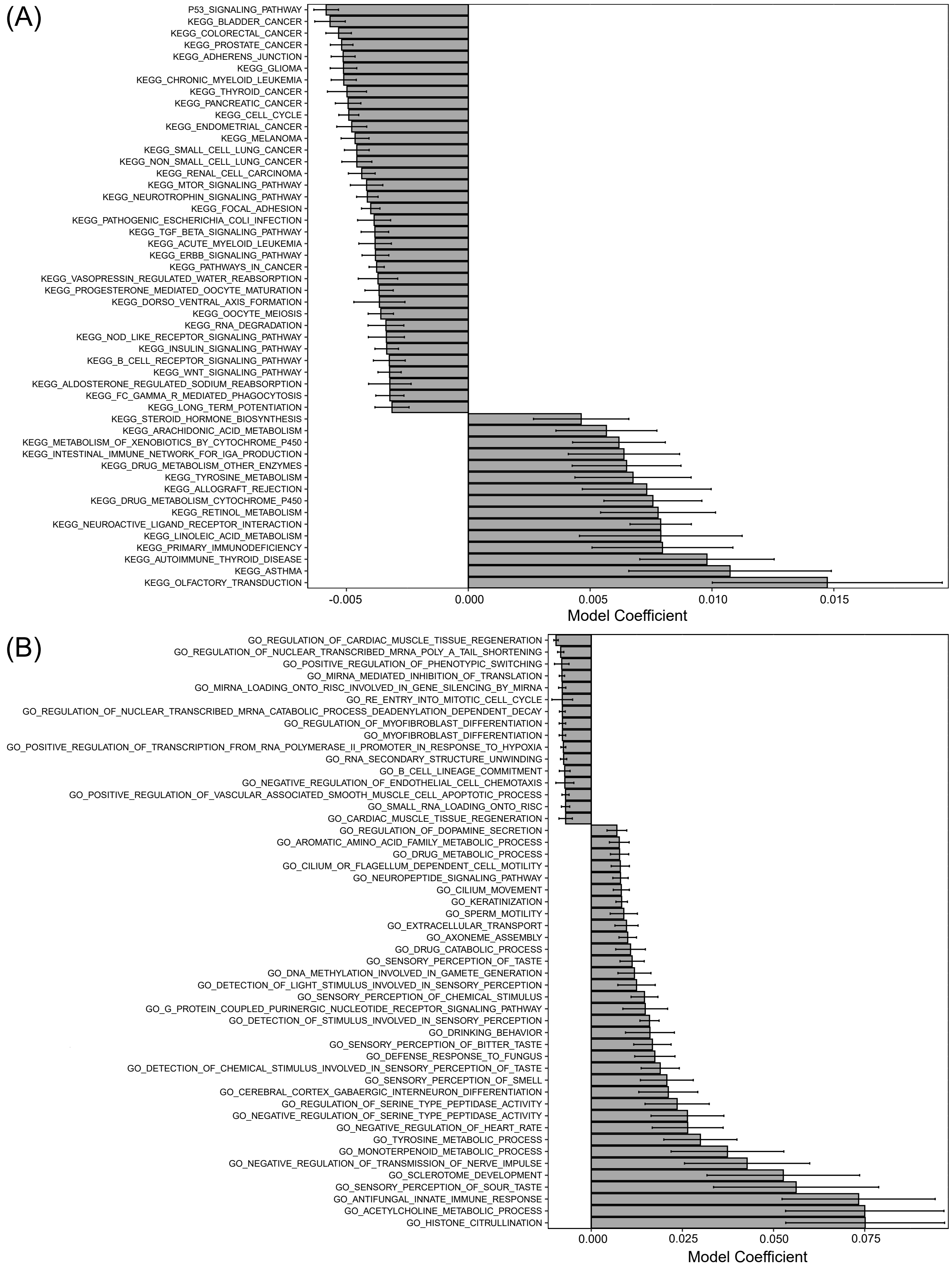

Supplementary Figure S2. Top 50 enriched gene sets in ovary vs testis from (A) KEGG and (B) Gene Ontology Biological Process databases. The bars are model coefficient ± standard error. Only the gene sets with FDR adjusted $p < 0.05$ are plotted. Generated using RBiomirGS R package.

Supplementary Figure S3

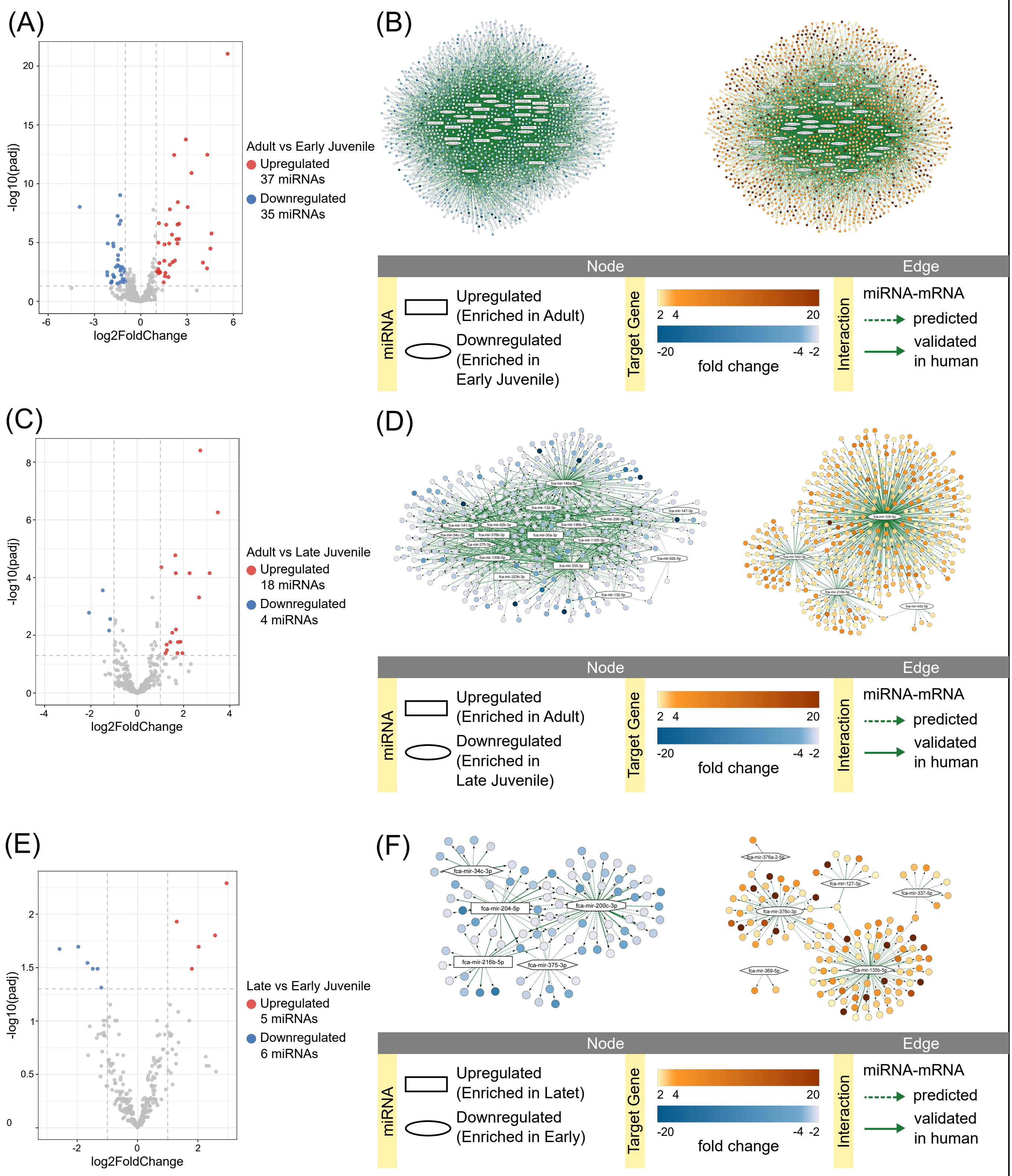

Supplementary Figure S3. miRNA expression in adult, early juvenile and late juvenile testicular tissue. (A) Volcano plot showing differentially expressed mature miRNAs in adult vs early juvenile testes. (B) miRNA-mRNA predicted interaction networks enriched in adult or early juvenile testes. (C) Volcano plot showing differentially expressed mature miRNAs in adult vs late juvenile testes. (D) miRNA-mRNA predicted interaction networks enriched in adult or late juvenile testes. (E) Volcano plot showing differentially expressed mature miRNAs in late vs early juvenile testes. (F) miRNA-mRNA predicted interaction networks enriched in late or early juvenile testes. Full size interactive networks are available in Supplementary Data Sheet S1.

Supplementary Figure S4

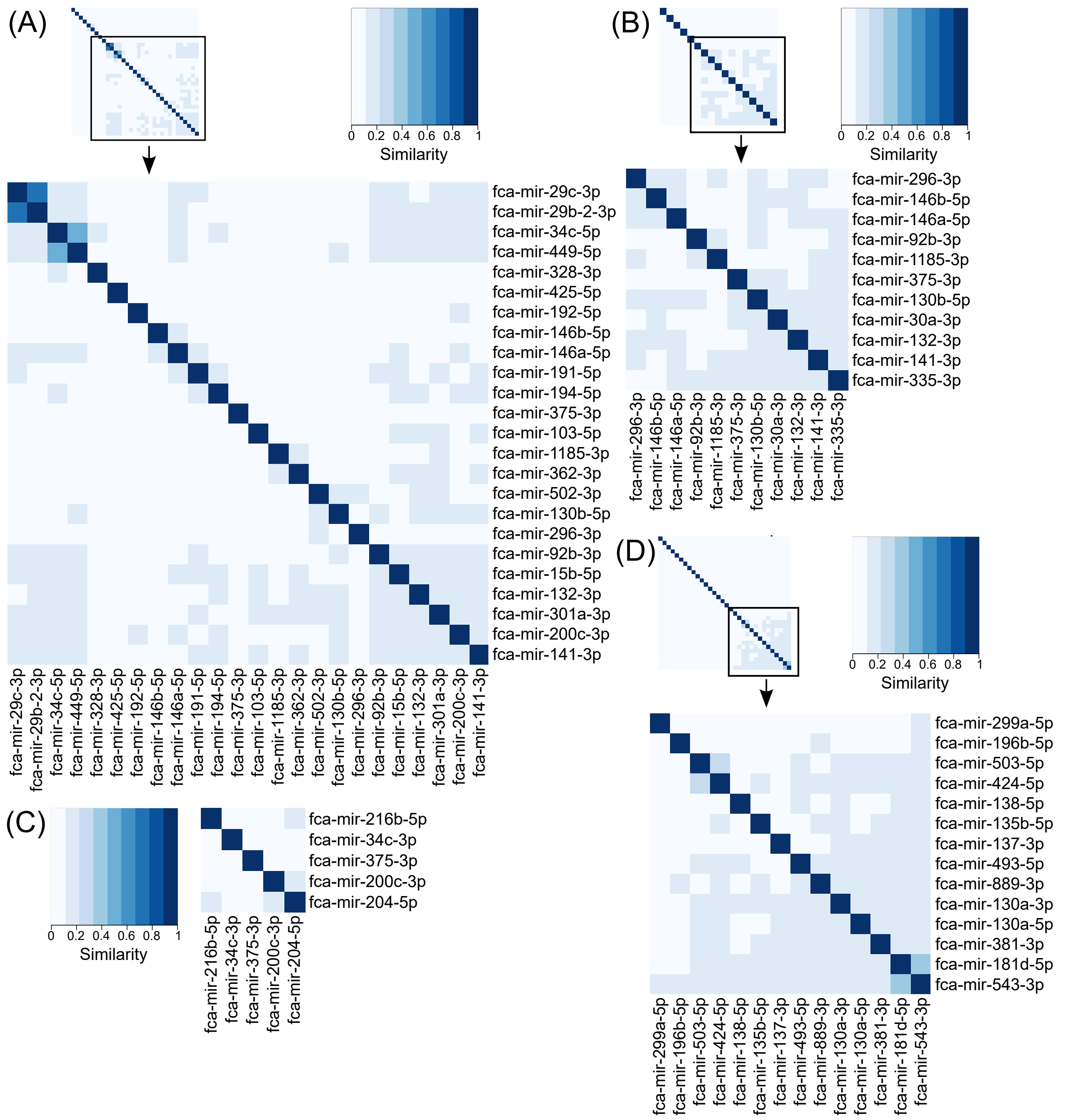

Supplementary Figure S4. Similarity of differentially expressed miRNA-mRNA predicted interactions in adult, early juvenile and late juvenile testes. Similarity heatmaps generated using miRmapper R package for upregulated miRNAs based on downregulated mRNA targets in (A) adult vs early juvenile, (B) adult vs late juvenile and (C) late vs early juvenile. (D) Similarity heatmap of downregulated miRNAs based on upregulated mRNA targets in adult vs early juvenile.
