# Supplementary material for: Feline microRNAome in ovary and testis: Exploration of in-silico miRNA-mRNA networks involved in gonadal function and cellular stress response
Source: Front Genet. 2022 Sep 26;13:1009220. doi: 10.3389/fgene.2022.1009220 (PMC9548565; doi:10.3389/fgene.2022.1009220)
Supplement: Supplementary file 4 [file Presentation1.zip › Supplementary Methods.docx]

**Supplementary Methods**

**Collection of ovarian and testicular tissues**

Ovaries and testes from prepubertal (3–6 months old) and testes from adult (over 1 year old) domestic cats were collected at local veterinary clinics as byproducts from owner-requested routine ovariohysterectomy or orchiectomy and transported in phosphate buffered saline (PBS) at 4°C to the laboratory within 6 h of excision. Ovarian cortical tissues were dissected into 1 x 1 x 0.2 mm pieces in dissection medium composed of minimum essential medium (MEM with Hank’s salt; Gibco Laboratories, Gaithersburg, MD) supplemented with 10 mM HEPES, 1 mM pyruvate, 2 mM L-glutamine, 100 IU/ml penicillin, 100 µg/ml streptomycin and 0.1% bovine serum albumin ^6^. Testes were washed with PBS, dissected from surrounding tissues and cut in pieces of approximately 2-3 mm^3^ in handling medium composed of Hepes-Ham’s F10 medium (Irvine Scientific, Santa Ana, CA) supplemented with 1 mM pyruvate, 2 mM L-glutamine, 100 IU/ml penicillin, 100 µg/ml streptomycin, 2.5% fetal bovine serum (FBS). For each animal, tissue pieces were either stabilized in RNAlater™ solution (Invitrogen, Carlsbad, CA) or processed for vitrification or dehydration as described below. All chemicals and reagents were purchased from Sigma-Aldrich (St. Louis, MO), unless otherwise indicated.

**Vitrification and warming of ovarian cortex**

Cortical pieces were threaded onto a 30-G needle (six pieces per needle, BD PrecisionGlide needle, Thermo Fisher Scientific, Waltham, MA) with space between, exposed to equilibration solution (7.5% dimethyl sulfoxide (DMSO) + 7.5% ethylene glycol (EG) + 30% FBS in base medium) for 10 min at 4°C followed by vitrification solution (15% DMSO + 15% EG + 20% FBS in base medium) for 10 min at 4°C, then plunged directly into liquid nitrogen and stored for at least 48 h in liquid nitrogen. Warming was performed by quickly transferring the needles to a sucrose gradient (1, 0.5, 0.25 and 0 M in base medium) for 5 min at each step at 37°C. Cortical pieces were then removed from the needles and incubated in RNAlater™ stabilization solution overnight at 4°C. After removal of stabilization solution, tissues were stored at -80°C until RNA isolation.

**Vitrification and warming of testicular tissues**

Tissue biopsies were exposed to equilibration solution (1.4 M DMSO + 1.4 M glycerol + 0.25 sucrose in Ham’s F10) for 10 min at room temperature followed by vitrification solution (2.8 M DMSO + 2.8 M glycerol + 0.5 M sucrose + 10% FBS in Ham’s F10) for 5 min at room temperature, then placed in cryotubes and plunged directly into liquid nitrogen and stored for at least one week in liquid nitrogen. Warming was performed by immersing cryotubes in a water bath at 50C for 5 s. Tissue fragments were then transferred to a sucrose gradient (0.5 M, 0.25 M and 0 M in Hepes-Ham’s F10 and 20% FBS) for 5 min at each step at room temperature. Tissues were incubated in RNAlater™ stabilizing solution and stored as described above.

**Microwave-assisted dehydration and rehydration of ovarian cortex**

Cortical pieces from the same ovaries used for vitrification were threaded onto a 30-G needle, immersed in in 10 µg/ml digitonin for 3 min to permeabilize the cell membrane, rinsed with dissection medium, incubated in 1M trehalose for 10 min and placed onto conjugate-release glass fiber filters (Whatman, Maidstone, UK). Samples were dehydrated in a SAM 255 microwave (CEM, Matthews, NC) for 5 or 10 min at 20% power (about 100 W power output) with upper temperature threshold set at 40°C. Dried cortical pieces were immediately rehydrated in dissection medium for 10 min at room temperature, then incubated in RNAlater™ stabilization solution and stored as described above.
